# Mimetic accuracy and co-evolution of mimetic traits in ant-mimicking species

**DOI:** 10.1016/j.isci.2022.105126

**Published:** 2022-09-14

**Authors:** Stano Pekár, Martina Martišová, Andrea Špalek Tóthová, Charles R. Haddad

**Affiliations:** 1 Department of Botany and Zoology, Faculty of Science, Masaryk University, Kotlářská 2, 611 37 Brno, Czech Republic; 2 Department of Zoology & Entomology, University of the Free State, P.O. Box 339, Bloemfontein 9300, South Africa

**Keywords:** Biological sciences, Evolutionary biology, Phylogenetics

## Abstract

Myrmecomorphy is the most frequent type of Batesian mimicry. Myrmecomorphic species differ in the accuracy with which they resemble ants; however, the hypothesis of the co-evolution of mimetic traits has been rarely tested. Here, we measured dozens of traits of color, shape, size, and behavior, and quantified objectively the resemblance between dozens of arthropod mimics and ants. In all traits, the mimics were more similar to ants than to closely related non-myrmecomorphic species. We found that mimics resemble ants mainly in color and behavior, and less in size and body shape. We found that the mimetic accuracy in four trait categories demonstrate divergent co-evolutionary patterns. Mimetic accuracy in color was positively correlated with shape and size in insects but negatively in spiders, presumably reflecting developmental constraints. Accuracy in shape tend to be negatively related to movement in both insects and spiders supporting the motion-limited discrimination hypothesis.

## Highlights

• We measured resemblance of ant mimics to ants in a number of arthropod species
• Mimics resembled ants mainly in color and behavior, less in size and body shape
• Ant mimics had similar behavior as ants but were smaller in body size
• Resemblance in shape was negatively related to resemblance in color in spiders

## Graphical abstract

**Figure:**
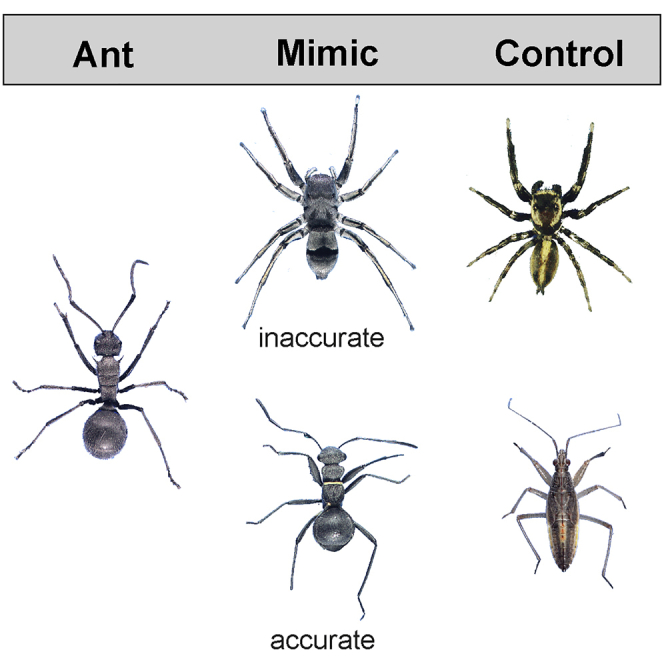

## Introduction

In Batesian mimicry, palatable mimics gain protection from generalist predators by imitating unprofitable models (Bates 1862). Batesian mimicry has been found in a variety of arthropod (e.g., hoverflies, butterflies, beetles) and vertebrate (e.g., fish, frogs, snakes, birds) taxa (e.g., Ruxton et al., 2019). Apparently the most successful type of Batesian mimicry is myrmecomorphy, or ant-mimicry, as it has been described in hundreds of arthropod species belonging to many different taxonomic groups (McIver and Stonedahl, 1993). Ants are frequent Batesian models not only because they possess a range of effective defenses, which make them unpalatable to generalist predators, but also because they are highly abundant and occur in a wide range of habitats (e.g., Hölldobler and Wilson, 1990). Unlike in other common Batesian mimics, such as butterflies or snakes, the majority of myrmecomorphic species imitate phylogenetically very distant models (McIver and Stonedahl, 1993; Pekár, 2022). Even in the absence of common ancestry, these mimics converged on the ant-like phenotype.

The degree of mimetic accuracy is variable in nature and depends on traits that mimic share with its model (e.g., Pekár, 2014,^2022^). In general, four mimetic categories of traits are recognized – color, shape, size, and behavior – which have been quantified in a few species only recently (e.g., Outomuro et al., 2016; Pekár et al., 2020; McLean and Herberstein, 2021). Even myrmecomorphic species range from very inaccurate to highly accurate mimics. Inaccurate mimics imitate only the color, whereas more accurate mimics also resemble ants in other traits (e.g., Moya-Laraño et al., 2013). The evolutionary histories of traits may differ because of different selection by the visually-oriented predators on each mimetic species. It is assumed that Batesian mimics are under constant selection for mimetic accuracy (e.g., Kikuchi and Pfennig, 2013). Although selection for an increased number of similar traits is expected (Kazemi et al., 2015), the prevailing modality of predators attacking the mimic should shape the mimetic phenotype (Pekár et al., 2011). Some traits (such as color) can simply overshadow other less salient traits (such as shape) (Kazemi et al., 2014,^2015^), thus relaxing selection for high mimetic accuracy in overshadowed traits. This may explain the proportionately high richness of inaccurate mimics (e.g., Kikuchi and Pfennig, 2013).

Relationships among mimetic traits can vary. One trait can compensate for poorer resemblance in another trait. For example, mimics matching the model color inaccurately could use behavior to compensate for their weaker ability to ‘blend in’ visually with their models (e.g., Pekár and Jarab, 2011; McLean and Herberstein, 2021). Alternately, accurate behavioral resemblance could be restricted to accurate morphological mimics (e.g., Nelson and Card, 2016), thus reflecting overall stronger selection for mimetic accuracy in all traits (Penney et al., 2014).

**Figure 1:**
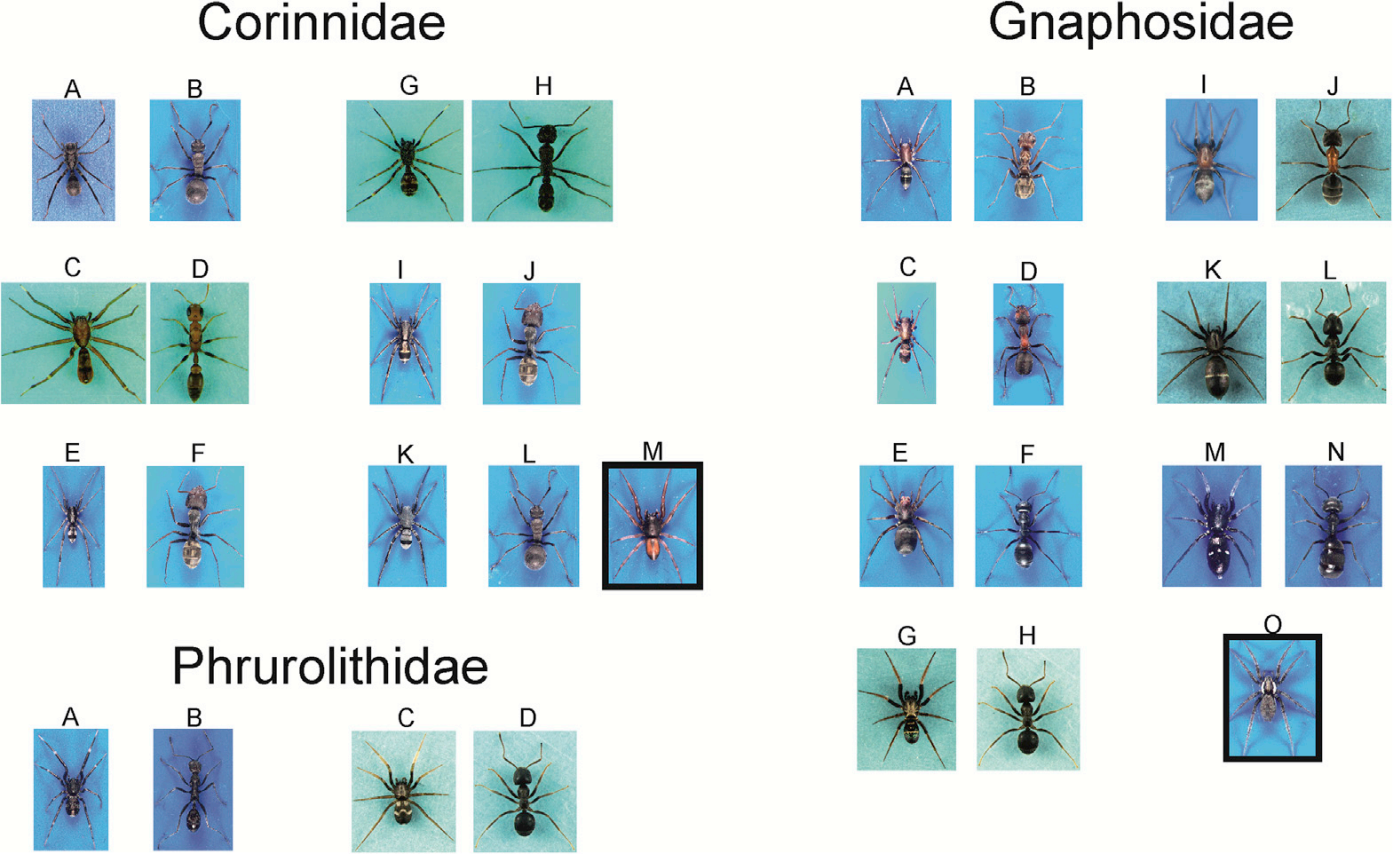
Habitus of study Corinnid, Gnaphosid and Phrurolithid mimic spiders (left columns), their ant models (right columns), and control species (in the box) Corinnidae: (A) *Apochinomma formicaforme*, B. *Polyrhachis schistacea*, C. *Castianeira rica*, D. *Pseudomyrmex* sp., E. *Castianeira* sp., F. *Camponotus cinctellus*, G. *Mazax pax*, H. *Ectatomma ruidum*, *Polyrhachis schistacea*, I. *Merenius alberti*, J. *Camponotus cinctellus*, K. *Corinnomma semiglabrum*, L. *Polyrhachis schistacea*, M. *Afroceto plana*. Gnaphosidae: A. *Micaria beaufortia*, B. *Anoplolepis custodiens*, C. *Micariaformicaria*, D. *Formica pratensis*, E. *M. fulgens*, F. *Formica fusca*, G. *M. micans*, H. *Lasius niger*, I. *M. sociabilis*, J. *Liometopum microcephalum*, K. *M. subopaca*, L. *Lasius platythorax*, M. *M. triguttata*, N. *Tapinoma erraticum*, O. *Asemesthes* sp. Phrurolithidae: A. *Liophrurillus flavitarsis*, B. *Aphaenogaster senilis*, C. *Phrurolithus festivus*, D. *Lasius niger*. Sizes are not to scale.

**Figure 2:**
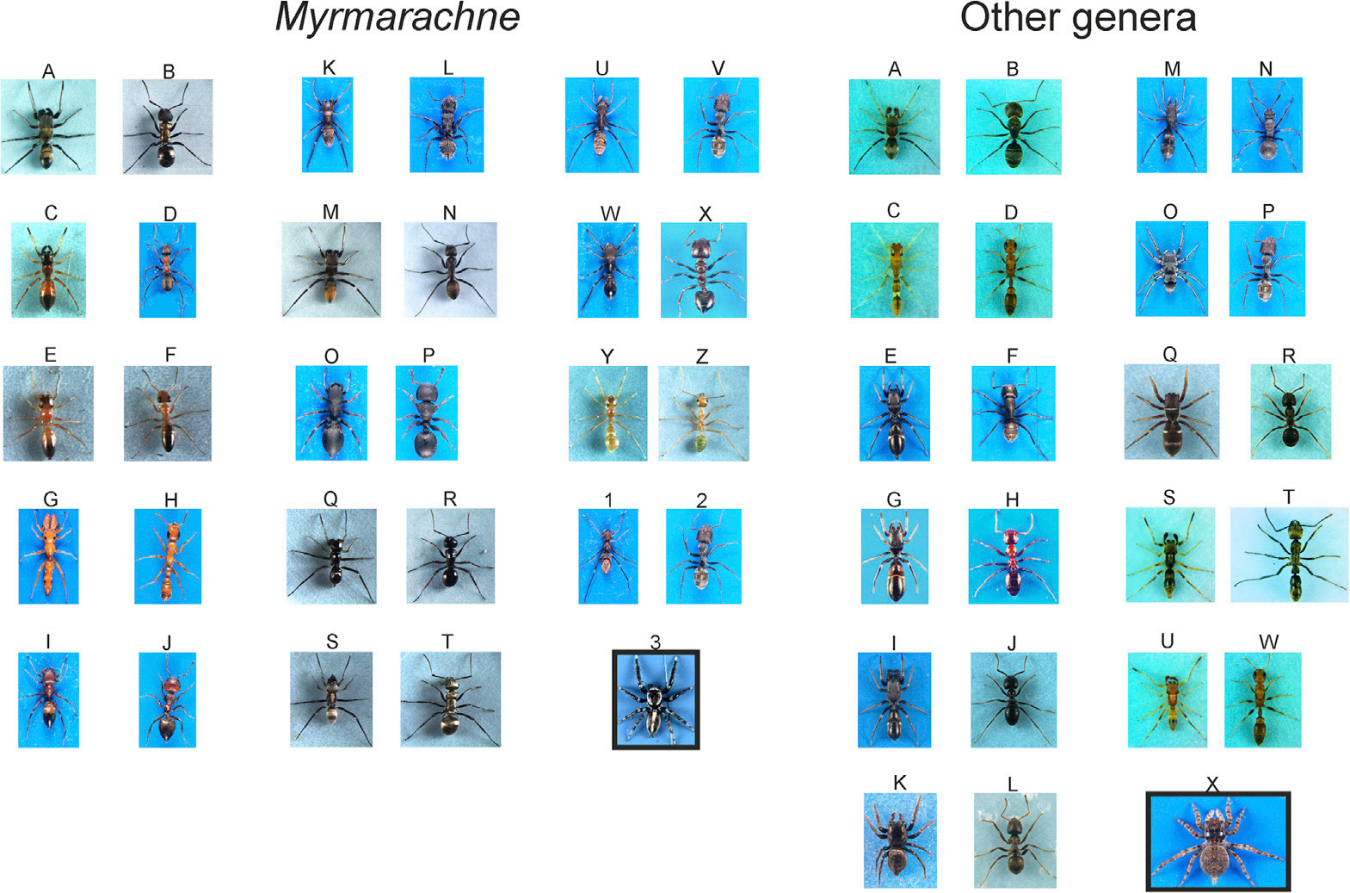
Habitus of study mimic salticid spiders (left columns), their ant models (right columns), and control species (in the box) *Myrmarachne*: A. *M. erythrocephala*, B. *Polyrhachis erato*, C. *M. formicaria*, D. *Formica rufibarbis*, E. *M. helensmithae*, F. *Opisthopsis haddoni*, G. *M. ichneumon*, H. *Tetraponera natalensis*, I. *M. kitale*, J. *Crematogaster castanea*, K. *M. laurentina*, L. *Camponotus postoculatus*, M. *M. luctuosa*, N. *Camponotus aeneopilosus*, O. *M. lulengana*, P. *Cataulacus intrudens*, Q. *M. macleayana robsoni*, R. *Polyrhachis robsoni*, S. *M. macleayana foreli*, T. *Polyrhachis foreli*, U. *M. marshalli*, V. *Camponotus cinctellus*, W. *M. russellsmithi*, X. *Crematogaster* sp., Y. *M. smaragdina*, Z. *Oecophylla smaragdina*, 1. *M. uvira*, 2. *C. cinctellus*, 3. *Phintelloides versicolor*. Other genera: A. *Corcovetella galianoae*, B. *Camponotus planatus*, C. *Synemosyna* sp., D. *Pseudomyrmex* sp., E. *L. berolinensis* – adult female, F. *Camponotus vagus*, G. *Leptorchestes berolinensis* - juvenile, H. *Colobopsis truncata*, I. *L. berolinensis* – adult male, J. *Lasius fuliginosus*, K. *Heliophanus flavipes*, L. *Lasius alienus*, M. *Kima variabilis*, N. *Polyrhachis schistacea*, O. *Mexcala elegans*, P. *Camponotus cinctellus*, Q. *Synageles venator*, R. *Lasius niger*, S. *Myrmapana costaricaensis* black, T. *Neoponera unidentata*, U. *Myrmapana costaricensis* brown, W. *Pseudomyrmex* sp. X. *Stenaelurillus* sp., Sizes are not to scale.

**Figure 3:**
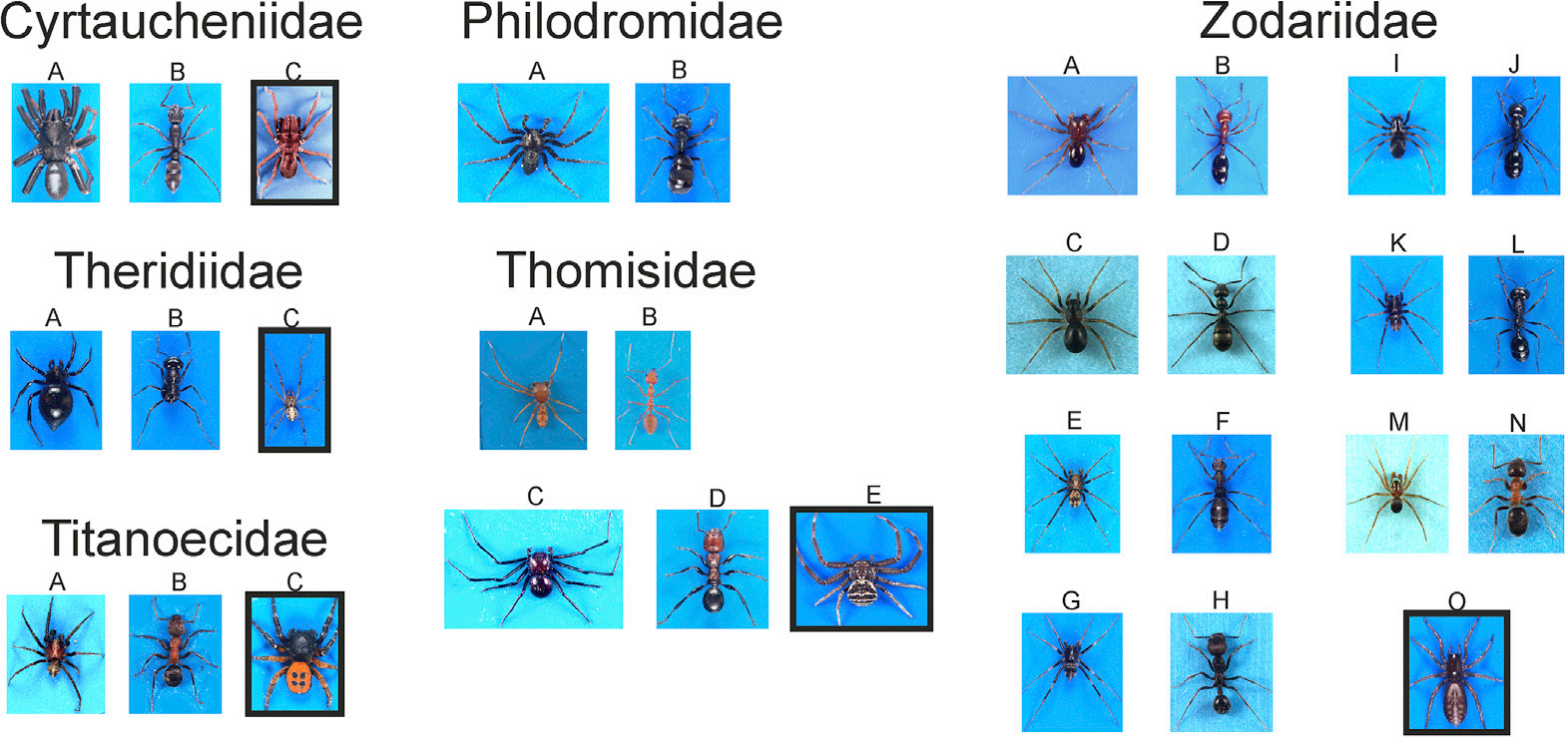
Habitus of study other mimic spiders (left columns), their ant models (right columns), and control species (in the box) Cyrtaucheniidae: A. *Ancylotrypa vryheidensis*, B. *Bothroponera kruegeri*, C. *Atypus affinis*. Theridiidae: A. *Euryopis episinoides*, B. *Messor barbarus*, C. *Phylloneta impressa*. Titanoecidae: A. *Titanoeca spominima*, B. *Formica sanguinea*, C. *Eresus kollari.*Philodromidae: A. *Pulchellodromus bistigma*, B. *Tapinoma erraticum*. Thomisidae: A. *Amyciaea* sp., B. *Oecophylla smaragdina*, C. *Sylligma ndumi*, D. *Atopomyrmex mocquerysi*, E. *Xysticus* sp. Zodariidae: A. *Trygetus sexoculatus*, B. *Monomorium venustum*, C. *Z. germanicum*, D. *Formica cinerea*, E. *Z. alacre*, F. *Iberoformica subrufa*, G. *Z. cyrenaicum*, H. *Messor arenarius*, I. *Z. luctuosum*, J. *Messor meridionalis*, K. *Z. nitidum*, L. *Messor dentatus*, M. *Z. rubidum*, N. *Lasius emarginatus*, O. *Selamia reticulata.* Sizes are not to scale.

**Figure 4:**
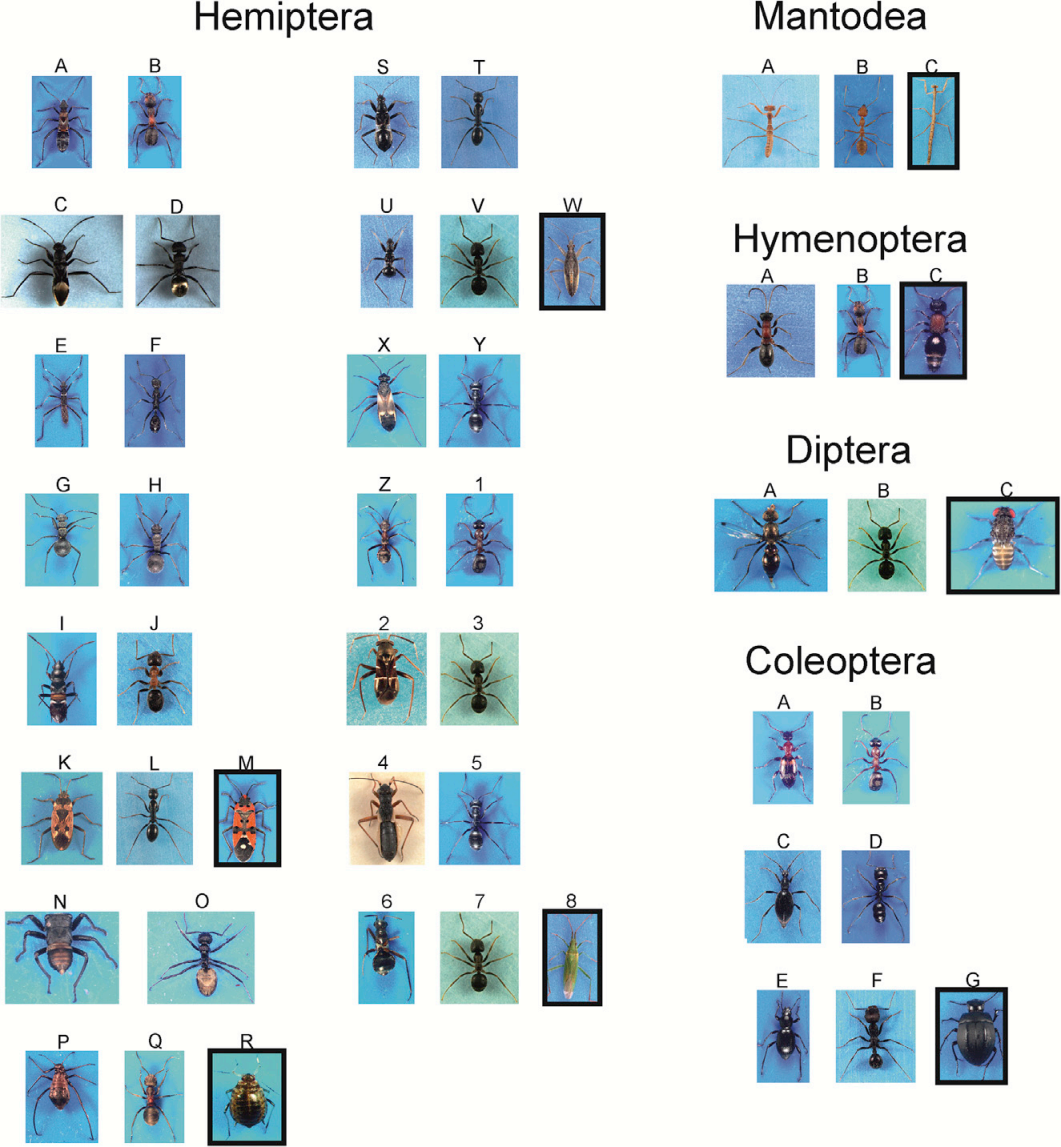
Habitus of study mimic insects (left columns), their ant models (right columns), and control species (in a box) Hemiptera: A. *Alydus calcaratus*, B. *Formica pratensis*, C. *Daerlac nigricans*, D. *Polyrhachis erato*, E. *Micrelytra fossularum*, F. *Aphaenogaster senilis*, G. *Myrmoplasta mira*, H. *Polyrhachis schistacea*, I. *Raglius alboacuminatus*, J. *Lasius emarginatus*, K. *Rhyparochromus vulgaris*, L. *Camponotus fallax*, M. *Lygaeus equestris*, N. *Eurymela rubrolimbata*, O. *Dolichoderus clarcki*, P. *Lachnus roboris*, Q. *Formica rufa*, R. *Myzus persicae*, S. *Himacerus mirmicoides* - big juvenile, T. *C. fallax*, U. *H. mirmicoides -* small juvenile, V. *Lasius niger*, W. *Nabis* sp., X. *Globiceps flavomaculatus*, Y. *Formica fusca*, Z. *Myrmecoris gracilis*, 1. *Formica cunicularia*, 2. *Pilophorus perplexus*, 3. *Lasius alienus*, 4. *Pithanus maerkelii*, 5. *Formica fusca*, 6. *Systellonotus triguttatus*, 7. *L. alienus*, 8. *Stenodoma* sp. Mantodea: A. *Sphodromantis lineola*, B. *Oecophylla smaragdina*, C. *M. formicaria*. Hymenoptera: A. *Gelis* sp., B. *Formica pratensis*, C. *Smicromyrme* sp. Diptera: A. *Sepsis thoracica*, B. *Lasius niger*, C. *Drosophila hydei.*Coleoptera: A. *Anthelephila pedestris*, B. *Formica cunicularia*, C. *Palaeostigus palpalis*, D. *Messor barbarus*, E. *Tentyrina orbiculata*, F. *Messor arenarius*, G. *Zophosis bicarinatus.* Sizes are not to scale.

Our aim in this study was to study patterns of co-evolution among mimetic traits. To this end we assessed accuracy of myrmecomorphy by measuring four categories of mimetic traits using taxonomically and geographically broad set of samples. For this purpose, we collected and measured four mimetic traits in more than 70 species (Table 1 and Figure 1, Figure 2, Figure 3, Figure 4) of ant-mimicking arthropods coming from almost all continents and belonging to a variety of taxonomic groups, namely spiders (Araneae), true bugs (Heteroptera), leafhoppers (Auchenorrhyncha), aphids (Sternorrhyncha), mantises (Mantodea), flies (Diptera), beetles (Coleoptera), and wasps (Apocrita).

**Table 1:** List of mimic and ant species used in the study, and the continent (site number) where they were collected, arranged by order and family (alphabetically) of mimics

Mimic (Order/Family/species-stage)	Ant (species)	Continent (site)
Araneae		
Corinnidae		
*Apochinomma formicaeforme* Pavesi^1^	*Polyrhachis schistacea* (Gerstäcker)	Africa (1)
*Castianeira rica* Reiskind^1^	*Pseudomyrmex* sp.	America (2)
*Castianeira* sp*.*^1^	*Camponotus cinctellus* (Gerstäcker)	Africa (3)
*Corinnomma semiglabrum* Simon^1^	*Polyrhachis schistacea*	Africa (1)
*Mazax pax* Reiskind^1^	*Ectatomma ruidum* (Roger)	America (2)
*Merenius alberti* Lessert^1^	*Camponotus cinctellus*	Africa (3)
Cyrtaucheniidae		
*Ancylotrypa vryheidensis* (Hewitt) - male^2^	*Bothroponera kruegeri* (Forel)	Africa (3)
Gnaphosidae		
*Micaria beaufortia* (Tucker)^3^	*Anoplolepis custodiens* (Smith)	Africa (4)
*M. formicaria* (Sundevall)^3^	*Formica pratensis* Retzius	Europe (5)
*M. fulgens* (Walckenaer)^3^	*Formica fusca* Linnaeus	Europe (6)
*M. micans* (Sundevall)^3^	*Lasius niger*	Europe (7)
*M. sociabilis* Kulczynski^3^	*Liometopum microcephalum* (Panzer)	Europe (8)
*M. subopaca* Westring^3^	*Lasius platythorax* Seifert	Europe (9)
*M. triguttata* Simon^3^	*Tapinoma erraticum* (Latreille)	Europe (10)
Philodromidae		
*Pulchellodromus bistigma* (Simon)^4^	*Tapinoma erraticum*	Europe (11)
Phrurolithidae		
*Liophrurillus flavitarsis* (Lucas)^1^	*Aphaenogaster senilis* Mayr	Europe (12)
*Phrurolithus festivus* (C. L. Koch)^1^	*Lasius niger* (Linnaeus)	Europe (13)
Salticidae		
*Corcovetella galianoae* Pekár^5^	*Camponotus planatus* Roger	America (2)
*Heliophanus flavipes* (Hahn)^5^	*Lasius platythorax*	Europe (15)
*Kima variabilis* Peckham & Peckham^5^	*Polyrhachis schistacea*	Africa (1)
*Leptorchestes berolinensis* (C. L. Koch)^5^	*Camponotus vagus* (Scopoli)	Europe (16)
*L. berolinensis* - juvenile^5^	*Colobopsis truncata* (Spinola)	Europe (16)
*L. berolinensis* - male^5^	*Lasius fuliginosus* (Latreille)	Europe (16)
*Mexcala elegans* Peckham & Peckham^5^	*Camponotus cinctellus*	Africa (17)
*Myrmapana costaricaensis* Pekár *-* black^6^	*Neoponera unidentata* Mayr	America (14)
*M. costaricensis* - brown^6^	*Pseudomyrmex* sp*.*	America (14)
*Myrmarachne erythrocephala* (L. Koch)^6^	*Polyrhachis erato* Forel	Australia (18)
*M. formicaria* (DeGeer)^6^	*Formica rufibarbis* Fabricius	Europe (19)
*M. helensmithae* Pekár^6^	*Opisthopsis haddoni* Emery	Australia (20)
*M. ichneumon* (Simon)^6^	*Tetraponera natalensis* (Smith)	Africa (17)
*M. kitale* Wanless^6^	*Crematogaster castanea* Smith	Africa (17)
*M. laurentina* Bacelar^6^	*Camponotus postoculatus* Forel	Africa (17)
*M. luctuosa* (L. Koch)^6^	*Camponotus aeneopilosus* Mayr	Australia (18)
*M. lulengana* Roewer^6^	*Cataulacus intrudens* (Smith)	Africa (17)
*M. macleayana foreli* (Bradley)^6^	*Polyrhachis foreli* Kohout	Australia (21)
*M. macleayana robsoni*^6^	*Polyrhachis robsoni* Kohout	Australia (21)
*M. marshalli* Peckham & Peckham^6^	*Camponotus cinctellus*	Africa (17)
*M. russellsmithi* Wanless^6^	*Crematogaster* sp*.*	Africa (17)
*M. smaragdina* Ceccarelli^6^	*Oecophylla smaragdina*	Australia (21)
*M. uvira* Wanless^6^	*Camponotus cinctellus*	Africa (17)
*Synageles venator* (Lucas)^5^	*Lasius niger*	Europe (22)
*Synemosyna* sp*.*^5^	*Pseudomyrmex* sp*.*	America (14)
Thomisidae		
*Amyciaea* sp.^4^	*Oecophylla smaragdina* (Fabricius)	Asia (23)
*Sylligma ndumi* Lewis & Dippenaar-Schoeman^4^	*Atopomyrmex mocquerysi* André	Africa (17)
Theridiidae		
*Euryopis episinoides* (Walckenaer)^7^	*Messor barbarus* (Linnaeus)	Europe (24)
Titanoecidae		
*Titanoeca spominima* (Taczanowski)^8^	*Formica sanguinea* Latreille	Europe (5)
Zodariidae		
*Trygetus sexoculatus* (O. Pickard-Cambridge)^9^	*Monomorium venustum* (Smith)	Africa (25)
*Zodarion alacre* (Simon)^9^	*Iberoformica subrufa* (Roger)	Europe (26)
*Z. cyrenaicum* Denis^9^	*Messor arenarius* (Fabricius)	Africa (27)
*Z. germanicum* (C. L. Koch)^9^	*Formica cinerea* Mayr	Europe (28)
*Z. luctuosum* (O. Pickard-Cambridge)^9^	*Messor meridionalis* (André)	Asia (29)
*Z. nitidum* (Audouin)^9^	*Messor dentatus* Santschi	Africa (25)
*Z. rubidum* Simon^9^	*Lasius emarginatus* (Olivier)	Europe (15)
**Mantodea**		
Mantidae		
*Sphodromantis lineola* (Burmeister) – 1st instar^10^	*Oecophylla smaragdina*	Africa (30)
**Hemiptera**		
Alydidae		
*Alydus calcaratus* (Linnaeus)^11^	*Formica pratensis*	Europe (5)
*Micrelytra fossularum* (Rossi)^11^	*Aphaenogaster senilis*	Europe (12)
Rhyparochromidae		
*Daerlac nigricans* Distant^11^	*Polyrhachis erato*	Australia (18)
*Raglius alboacuminatus* (Goeze)^11^	*Lasius emarginatus*	Europe (31)
*Rhyparochromus vulgaris* (Schilling)^11^	*Camponotus fallax* (Nylander)	Europe (32)
Miridae		
*Globiceps flavomaculatus* (Fabricius)^12^	*Formica fusca* Linnaeus	Europe (31)
*Myrmecoris gracilis* (Sahlberg)^12^	*Formica cunicularia*	Europe (5)
*Pilophorus perplexus* Douglas & Scott^12^	*Lasius alienus*	Europe (32)
*Pithanus maerkelii* (Herrich-Schaeffer)^12^	*Formica fusca*	Europe
*Systellonotus triguttatus* (Linnaeus)^12^	*Lasius alienus*	Europe (16)
Nabidae		
*Himacerus mirmicoides* (O. G. Costa) - big juvenile^13^	*Camponotus fallax*	Europe (31)
*H. mirmicoides -* small juvenile^13^	*Lasius niger*	Europe (31)
Pyrrhocoridae		
*Myrmoplasta mira* Gerstäcker^13^	*Polyrhachis schistacea*	Africa (1)
Aphididae		
*Lachnus roboris* (Linnaeus)^14^	*Formica rufa* Linnaeus	Europe (32)
Cicadellidae		
*Eurymela rubrolimbata* Kirkaldy^14^	*Dolichoderus clarcki* Wheeler	Australia (33)
**Coleoptera**		
Anthicidae		
*Anthelephila pedestris* (Rossi)^15^	*Formica cunicularia* Latreille	Europe (7)
Staphylinidae		
*Palaeostigus palpalis* (Latreille)^15^	*Messor barbarus*	Europe (26)
Tenebrionidae		
*Tentyrina orbiculata* (Fabricius)^15^	*Messor arenarius*	Europe (27)
**Hymenoptera**		
Ichneumonidae		
*Gelis* sp*.*^16^	*Formica pratensis*	Europe (31)
**Diptera**		
Sepsidae		
*Sepsis thoracica* (Robineau-Desvoidy)^17^	*Lasius niger*	Europe (34)

Unless otherwise stated, adult females were used. See Table S2 for site descriptions. The following non-myrmecomorphic (control) species (superscript) were used: ^1^*Afroceto plana* Lyle & Haddad (Corinnidae), ^2^*Atypus affinis* Eichwald (Atypidae), ^3^*Asemesthes ceresicola* Tucker (Gnaphosidae), ^4^*Xysticus* sp. (Thomisidae), ^5^*Phintelloides versicolor* (C. L. Koch) (Salticidae), ^6^*Stenaelurillus* sp. (Salticidae), ^7^*Phylloneta impressa* (L. Koch) (Theridiidae), ^8^*Eresus kollari* Rossi (Eresidae), ^9^*Selamia reticulata* (Simon) (Zodariidae), ^10^*Danuria* sp. (Mantodea), ^11^*Lygaeus equestris* Linnaeus (Lygaeidae), ^12^*Stenodema* sp. (Miridae), ^13^*Nabis* sp. (Nabidae), ^14^*Myzus persicae* (Sulzer) (Aphididae), ^15^*Zophosis bicarinata* Solier (Coleoptera), ^16^*Smicromyrme* sp. (Mutillidae), ^17^*Drosophila hydei* Sturtevant (Diptera).

## Results

### Accuracy of trait similarity

#### Color

Mimics were more similar to ants in color than to control species. There was a significant difference in the reflectances (Figures S1 and S2) of bodies between mimic-ant and mimic-control pairs (LME, F_1,139_ = 161.1, p < 0.0001, Figures S3A and S4).

#### Shape

Mimics had significantly less elongated and less articulated bodies than ants, but more elongated and articulated bodies than controls (Figure S5), according to significant differences in body roundness (LME, F_2,1298_ = 341, p < 0.0001). The group random effect in body roundness explained much less variation than the triplet effect (16.2 vs. 29.4%), so there was a considerable difference among triplets (mimic-ant-control) within each taxonomic group (Figure S6A). However, mimics were less similar in body outline to ants than to controls, as there was a significant difference in the Euclidean distances of outline between mimic-ant and mimic-control pairs (LME, F_1,139_ = 29.4, p < 0.0001, Figures S3B and S6B).

#### Size

**Figure 5:**
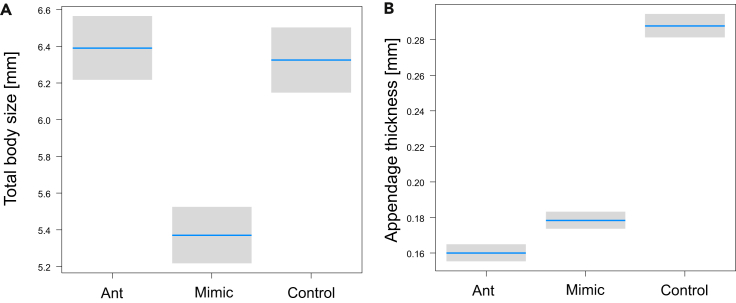
Mimics are not similar to ants in body size but in appendage thickness Comparison of total boy size (A) and appendage thickness (B) among mimics, ants and control species. Blue lines are estimated means, gray bars are 95% confidence intervals.

There were significant differences among mimics, ants, and controls in size traits (MANOVA, Pillai _21,298_ = 1.69, p < 0.0001). Total body length was significantly different (LME, F_2,1298_ = 120.6, p < 0.0001): mimics were significantly shorter than ants and controls (Figure 5A). The group random effect in body length explained much less variation (45.9 vs. 10%), so there was a considerable difference among triplets within each taxonomic group (Figure S7A). The thickness of appendages was significantly different (LME, F_2,1298_ = 503.2, p < 0.0001): mimics had significantly thicker appendages than ants but significantly thinner than controls (Figure 5B). The groups explained less variation (35.6 vs. 19.5%); thus, there were considerable differences among triplets within each taxonomic group (Figure S7B).

#### Movement

**Figure 6:**
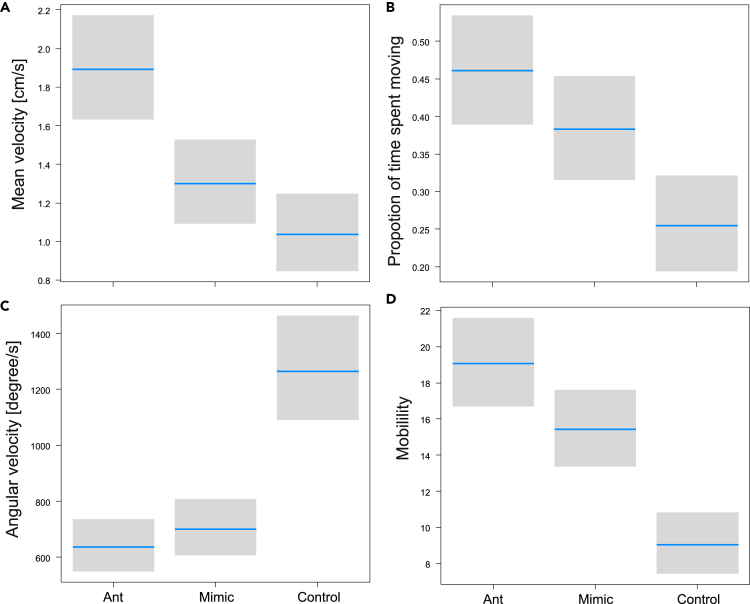
Mimics are similar to ants in movement Comparison of velocity (A), time spent moving (B), angular velocity (C) and mobility (D) among mimics, ants and control species. Blue lines are estimated means, gray bars are 95% confidence intervals.

There were significant differences in movement traits (MANOVA, Pillai _21,456_ = 0.36, p < 0.0001). The mean velocity was significantly different (LME, F_2,1388_ = 133.5, p < 0.0001): mimics were slightly slower than ants but significantly faster than controls (Figure 6A). The groups explained slightly less variation than the effect of triplet (22 versus 24%), thus, a few triplets in each taxonomic group showed a different pattern (Figure S8A). The proportion of locomotion was significantly different (LME, F_2,1388_ = 115.9, p < 0.0001): mimics spent less time moving around than ants but more time than controls (Figure 6B). The groups explained a similar proportion of variation (22% each), thus, there were similar differences among triplets within each group as among groups (Figure S8B). The angular velocity was significantly different (LME, F_2,1388_ = 316.3, p < 0.0001): mimics exhibited slightly higher angular velocity than ants, but significantly lower than controls (Figure 6C). The groups explained less variation (15 vs. 20%), thus, there were considerable differences among triplets within each group (Figure S8C). The mobility was significantly different (LME, F_2,1388_ = 219.8, p < 0.0001): mimics performed slightly fewer movements than ants but significantly more than controls (Figure 6D). The groups explained less variation (22 vs. 25%), thus, there were considerable differences among triplets within each group (Figure S8D).

### Trait co-evolution

**Figure 7:**
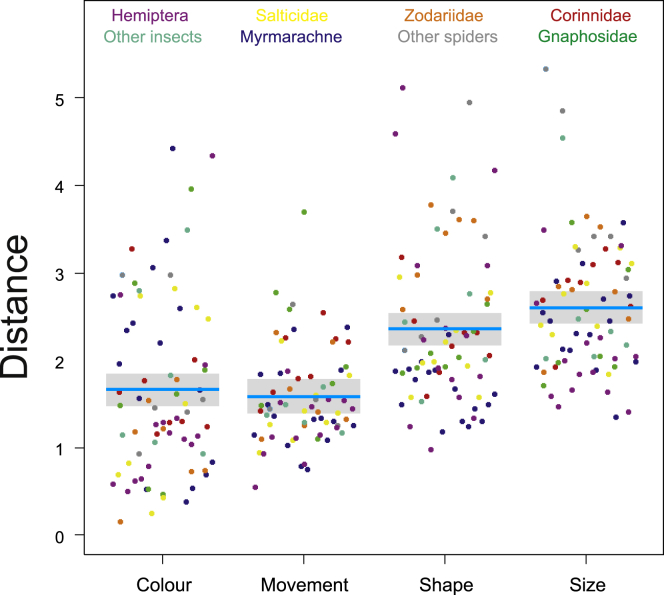
Mimics resemble ants more in color and movement than in shape and size Comparison of mimic-model accuracy (Euclidean distance) among four categories of mimetic traits. Blue lines are estimated means, gray bars are 95% confidence intervals of the mean.

**Figure 8:**
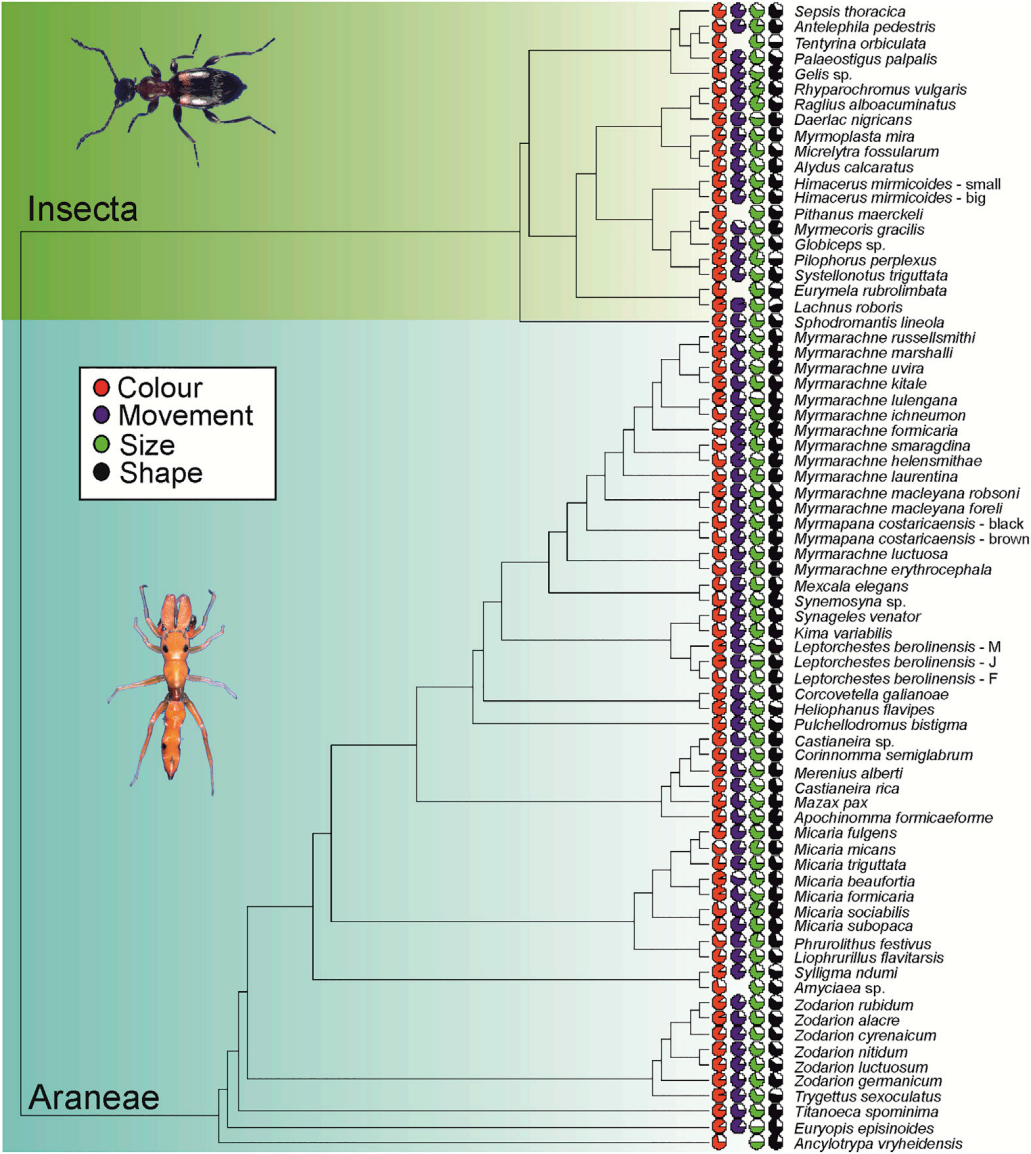
Phylogenetic signal was weak to moderate for mimetic traits Phylogenetic tree of all mimetic species studied with estimates of their mimetic accuracy in four trait groups. The fuller the circle, the higher the relative mimetic accuracy in the particular trait category.

We estimated mimetic accuracy for each mimic-ant pair in all four trait categories. There were significant differences among four trait categories (LME, F_3,214_ = 29.6, p < 0.0001): mimics were most similar to ants in movement and color, followed by shape and size (Figure 7). Then we mapped the estimates of mimetic accuracy on a phylogenetic tree of mimics. We found the phylogenetic signal of accuracy of mimetic traits to be weak for color (GLS, Pagel’s λ = −0.02), low for movement (λ = 0.30) and shape (λ = 0.36), and moderate for size (λ = 0.48, Figure 8).

**Figure 9:**
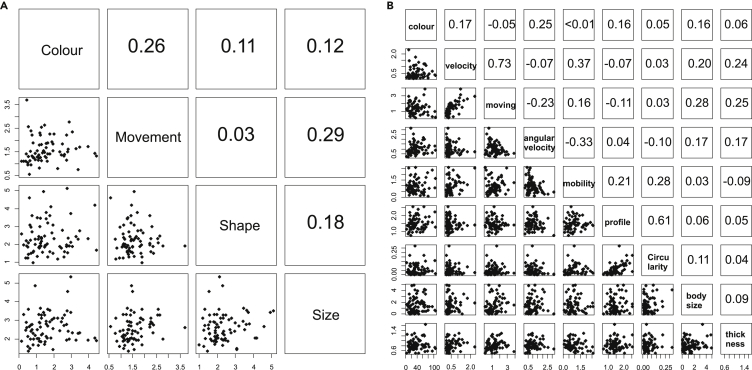
Mimetic traits were overall weakly correlated Correlation matrix of Euclidean distances of the four mimetic trait categories (A) and all mimetic traits (B) with estimated Pearson correlations.

Correlations between the accuracy (measured as distances) of four mimetic trait categories were all positive but not statistically significant (GLS, p > 0.06, Figure 9A). Correlations between accuracy of all mimetic traits were not statistically significant (GLS, p > 0.05, Figure 9B).

**Figure 10:**
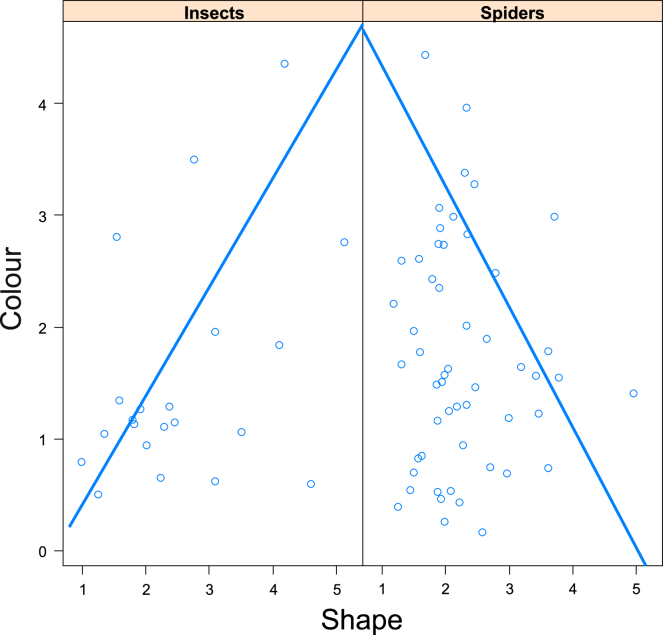
Co-evolution of mimetic trait differences between spider and insect mimics Relationships between mimetic accuracy (Euclidean distance) of color and shape split by two taxonomic groups, insects and spiders. Linear models were estimated using GLS with a phylogenetic covariance matrix. Blue lines are estimated linear models.

There were, however, significant relationships when the relationships among four mimetic trait categories were studied separately for two taxonomic groups, insects and spiders. Specifically, color accuracy was not significantly related to movement either for insects or spiders (GLS, F_1,65_ = 3.0, p = 0.089), but color was significantly positively related to shape in insects and negatively to shape in spiders (GLS, F_1,70_ = 13.0, p = 0.0006, λ = 0.09, Figure 10). Color was also positively related to size in insects and negatively to size in spiders but not significantly after Bonferroni correction (GLS, F_1,70_ = 4.4, p = 0.04, λ = 0.05). Size was not significantly related to movement in both insects and spiders (GLS, F_1,66_ = 2.6, p = 0.11). Shape was not significantly related to size either in insects or spiders (GLS, F_1,70_ = 0.7, p = 0.41), neither to movement in insects and spiders after Bonferroni correction (GLS, F_1,65_ = 5.3, p = 0.024, λ = 0.85). There was no significant relationship between color accuracy and the velocity or time spent moving (GLS, F_1,65_< 0.1, p > 0.68).

## Discussion

In this study, we performed multi-trait analysis of accuracy in more than 70 myrmecomorphic species and obtained thousands of measurements. We objectively and precisely quantified similarities among mimic, ant, and control species in a number of mimetic traits. The species in triplets (mimic, model, and control) were selected based on perceived (by humans) similarity/difference in coloration, shape, and size; thus, it may seem trivial to find mimics be similar to models than to controls. However, the perceived similarity was superficial, which can differ from exact measurements performed. Indeed, we found that in size the mimics and models are not similar.

Similar multi-trait analyses of accuracy in Batesian mimics are scarce and have been performed only very recently and on very few species (e.g., Malcicka et al., 2015; Hashimoto et al., 2020; Pekár et al., 2020; McLean and Herberstein, 2021; Kelly et al., 2021). In the past, analyses were either subjective or based only on a few trait categories, such as color alone (e.g., Holloway et al., 2002,; Corcobado et al., 2016) or color and shape (e.g., Raška and Pekár, 2019; McLean and Herberstein, 2021).

We found that mimics imitate ants more by color and movement and less by shape and size. This distinction corresponds to the level of phenotypic plasticity in these traits: highest for behavioral and color traits and lowest for morphological traits. Yet size should be more plastic than shape, because it is influenced by the amount of resources available during ontogenetic development (but see below). The mimetic signal that predators perceive is very likely multi-component, although not all traits are equally significant (Franks and Sherratt, 2007). Predators may use information only from one or a few traits (Sherratt et al., 2015). A high salience trait could overshadow less salient traits (Kazemi et al., 2014,^2015^). Should this be true, then the selection for lower salience traits should be weaker than for high salience traits, resulting in lower similarity in some traits. Our results are consistent with results of former experiments, which revealed that the color of an object has about a three times higher salience than size and shape for bird and human observers (Lazareva et al., 2005; Kazemi et al., 2014).

Although the body shape of ant-mimics was more elongated and articulated (as indicated by body roundness) than that of control species, the body outline of mimics was more similar to the control species. This is because of the different positions of constrictions (separating the head, thorax, and gaster) between mimics and ants. This could be explained by developmental and genetic constraints (Holloway et al., 2002). According to this hypothesis, mimics cannot achieve a high degree of accuracy because of insufficient genetic variation, which could be overcome by the passing of sufficient evolutionary time in combination with strong selection (Kikuchi and Pfennig, 2013).

It is, however, not known whether predators can distinguish such minute shape differences. For example, birds, as potentially one of the most important selective agents, can distinguish tiny differences in the morphology of insects, such as the shape of antennae (e.g., Bain et al., 2007) and can thus distinguish an object when stationary. However, while moving, the elongated shape might be more important than the position of constrictions. As for other potential predators, even some spiders or mantises, should be able to perceive small shape differences. Similarly, it is not known whether predators could distinguish small but significant differences in sizes. Predators could take a ‘gestalt’ approach where combinations of traits are more important than any individual trait.

Of interest, mimics were of a smaller size than both ants and control species. Yet, mimics that imitate polymorphic ant species resemble the smallest morphs. There should be a positive selection for larger body size not only because of the improved resemblance to models but also because of increased fecundity, which is a function of body size (e.g., Honěk, 1993; Head, 1995). The smaller body size of mimics could result from antagonistic selection – for example, by specialized predators. Ant-eating predators select larger ant workers (e.g., Pekár et al., 2017), presumably because of their higher profitability compared to smaller ones. The bodies of ants are generally slender and a larger body size should provide higher energetic and nutritional benefits (Pekár and Mayntz, 2014). By resembling small ant morphs, mimics may avoid predation by both generalist and specialized ant-eating predators.

The other measure of size, appendage thickness, showed a completely different pattern – mimics resembled ants more than control species. This shows that selection forces differ even within the same category of traits. Slender appendages, similarly to an elongated body, should be beneficial in improving resemblance to the locomotion of ants, such appendages allowing faster locomotion (e.g., Brandt and Andrade, 2007), in which mimics are very similar to their models.

Overall, the phylogenetic signal of the study trait categories was rather weak and different among traits, which is common when various traits (morphological and behavioral) are compared (e.g., Kamilar and Cooper, 2013). This is not surprising because ant-mimicry is a response to local ecological conditions and traits like coloration or movement are plastic and not shared among closely-related species (Pekár 2014). In addition, ant mimicry has evolved independently in many genera of arthropods, spread across many families (McIver and Stonedahl, 1993). However, other traits, namely body shape or body size are shared and indeed these traits showed higher phylogenetic signal.

Our data reveal weak overall correlations among the four categories of traits, suggesting divergent co-evolutionary patterns conditioned at least by taxonomy. Positive co-evolution among traits should result in more successful protection from predators (Kazemi et al., 2015). However, a constraint on one mimetic signal could be compensated by the improvement of another one (Kilner et al., 1999). The observed contrasting relationship between color and shape for insects and spiders shows that in insects with a body plan very similar to that of ants the evolution of color similarity is paralleled, whereas in spiders color appears to compensate for differences in shape. Similar relationships were found for color and size, although not significantly; thus, in insects, the three mimetic trait categories (color, shape, and size) appear to show positive co-evolution, whereas in spiders color might compensates for lower accuracy in shape and size. However, the strength of the relationship is weak suggesting that the co-evolution and compensation mechanisms can be species specific.

We found some support for compensation between movement and shape traits. According to the motion-limited discrimination hypothesis, inaccurate mimics would move faster and spend more time moving than accurate mimics to reduce assessment of their mimetic accuracy by predators (McLean and Herberstein, 2021). Although Penney et al. (2014) found behavioral mimicry occur in accurate mimics in hoverflies, McLean and Herberstein (2021) failed to find a correlation between morphology and speed of locomotion or time spent in locomotion in several ant-mimicking spiders and insects from Australia. The lack of compensation between color accuracy and movement could be because predators could perceive color of moving mimics more than their body shape. This is line with very recent results: An artificial increase in the locomotion speed of mimics reduced the probability of correct identification by human observers, suggesting room for compensation between speed and other mimetic traits (Pekár, 2021).

In our study, we used linear methods to assess mimetic resemblance. Ideally, the resemblance should be based on the perception and response of potential predators because the mimicry is ‘in the eye of the beholder’ (Cuthill and Bennett, 1993). This is not feasible, primarily because of the broad array of mimetic species used here where each can be under selection from a different local predator community. Second, the perception (visual) models have been developed only for a single trait, the coloration, or few traits, coloration and morphology, so far.

We conclude that ant-mimicking arthropods resemble their ant models in a number of traits, but mainly in color and movement. Given the multi-trait signaling to potential predators, the role of mimetic traits may differ not only because of genetic constraints but also because of different selection pressures exerted by the local community of predators.

### Limitations of the study

We recognize the following limitations: (1) Taxon sampling was not optimal as we failed to include more mantids, thrips, beetles, and other insect ant-mimics (McIver and Stonedahl, 1993); (2) species sampling was not optimal either geographically, as we failed to include many central African and Asian ant-mimics; (3) we failed to measure body shape of mimics/models from a lateral side which should be important angle of view for small arthropods predators. Experiments with different predators would be needed to address the salience of different mimetic traits. Future investigation that would record ecological variables, such as habitat type, are needed to reveal under what conditions ant-mimicking phenotypes are favored (Pekár 2022).

## STAR★Methods

### Key resources table

**Table:** 

REAGENT or RESOURCE	SOURCE	IDENTIFIER
**Deposited data**		

Raw and analyzed data	This paper	https://doi.org/10.17632/k4rb7xgry9.1

**Software and algorithms**		

R	R Core Team (2021)	https://www.R-project.org/.

### Resource availability

#### Lead contact

Further information and requests for resources should be directed to and will be fulfilled by the lead contact, Stano Pekár (pekar@sci.muni.cz).

#### Materials availability

This study did not generate new unique reagents.

### Experimental model and subject details

#### Species

At each of 34 study sites (Table S1) where a mimetic species was found, we investigated co-occurring ant species to select a putative model on the basis of its similarity in circadian activity, coloration, and approximate body size. If more than one ant species of a very similar phenotype co-occurred then we selected the one that was more abundant as a putative model. Only in a few cases, the model had already been reported (Edmunds, 1976; Deckert and Wachmann, 2020; Pekár, 2022), although this was often subjective. As non-myrmecomorphic species (henceforth referred to as control), we used closely related representatives from each family or order, altogether 17 species (Table 1 and Figure 1, Figure 2, Figure 3, Figure 4). As it was not always possible to find co-occurring control species, we used others either from different habitats or laboratory cultures.

Several mimetic species appear to resemble the same ant model (Table 1). This was mainly the case of species from Australia and South Africa, where they form mimetic complexes. In such cases we used different set of measurements of a model species when paired with a different mimic. Similarly, the same control species was used for many triplets, however, with different set of measurements.

Then, we collected 2–15 specimens of mimic species, 7–17 specimens of the putative ant model (Figure 1, Figure 2, Figure 3, Figure 4), and 6–13 specimens of control species. Mimetic species and their ant models, as well as control species, were collected by means of various sampling methods depending on the microhabitat type: The sweeping of low vegetation; the beating of tree foliage; and hand sampling on the ground, on tree trunks, and in leaf litter. Mimetic and control specimens were placed singly in Eppendorf tubes with a punctured lid and brought to the laboratory alive. Ant workers from a single nest were placed in polyethylene jars (200 mL) with a piece of wet tissue and brought to the laboratory. In the majority of species, only a single sex/developmental stage was used, but in two species (*Himacerus mirmecoides*, *Leptorchestes berolinensis*) various developmental stages/sexes were used because of their resemblance to different ant models.

### Method details

#### Phenotypic similarity

We quantified the behavior, body size, body shape, and color. In five mimetic species (*Ancylotrypa vryheidensis*, *Amyciaea* sp., *Eurymela rubrolimbata*, *Pithanus maerkelii*, and *Tentyrina orbiculata*) we failed to record their behavior. Of the four trait categories, only the behavior was measured in the field using the same instruments (see below).

The behavioral components should include seasonal and circadian activity, locomotion, and body movements, such as mobility, abdomen bobbing, and foreleg/antennae waving (e.g., Penney et al., 2014). Because we selected putative models among ants, which were seasonally and diurnally active with their mimics, seasonal and circadian activity were not measured. Two of the body movements (abdomen bobbing and foreleg/antennae waving) were excluded from the analysis, because the former occurred in only a very few species (only *Castianeira*, *Micaria*, and *Tapinoma* species) and the other in all species. Thus, we only focused on movement traits.

We first recorded the movement (locomotion and body motion) using a video camera (Canon Legria HF R606, 30 fps). We placed an individual into a white plastic container. To prevent its escape, we applied a very thin film of butter onto the interior walls. The film did not alter the behavior of ants, mimics or control species. We used two different containers: large specimens were placed into a larger rectangular container (19 × 27 × 10 cm) and small specimens were placed into a smaller rectangular container (14 × 19 × 5 cm). The same size of container was used for any given triplet (mimic, model, control). The containers were a thousand times larger than the arthropod, thus providing sufficient space for unconstrained locomotion. We recorded the continuous movement of each specimen for a few minutes. Then we selected a 1-min long piece of footage of its locomotion once the specimen had settled down. The container was cleaned with hot water and dried after each trial (e.g., Pfeifer et al., 2010). The video recordings were then processed using EthoVision XT software (Noldus Information Technology) to quantify the movement. We estimated four traits for each specimen: the mean velocity (cm/s), which described how quickly it moved; mobility (i.e., percentage of time that body moved while stationary), which described body movement (body turning, orientation); locomotion (s) (i.e., the cumulative duration of moving), which described how often it moved in space; and absolute angular velocity (degree/s), which described the path shape.

Then, we preserved the arthropods by exposing them to ethyl acetate for a few minutes and placed them on a piece of black paper to measure their reflectance using an Ocean Optics USB4000 spectrometer connected to an Ocean Optics PX-2 pulsed Xenon light source, which emits light in the range of 220–750 nm. The probe was a Y-bifurcated fiber. The reflectance values (300–700 nm) were relative to a white standard (PTFE optical diffuser reflecting >98% along the entire wavelength range, Ocean Optics WS-1). A black standard was obtained by pointing the fibers at the piece of black paper. The optical fiber was 400 nm in diameter and was held 1 cm above the subject, positioned above at a vertical 60° angle. We took two measurements of the whole body, one focused on the whole anterior body part and the other on the whole posterior body part (Figures S1 and S2). Thus, for each specimen, we obtained 800 values (400 for each body part).

Specimens were then mounted on blue sticky tape in a natural position. The blue color was used to allow the software to extract the image mask more easily. We took three pictures of every individual in dorsal view using an Olympus X12 stereomicroscope with an Olympus SC50 camera; these pictures were then combined into one image using Olympus Stream Motion software. We illuminated specimens from the side (approximately at 45°) by means of two fluorescent bulbs (13-W daylight Repti Glo 2.0 UVB) with a similar light spectrum to natural light. We then analyzed the images by means of custom-made image analysis software (Ježek, 2015) to obtain data on sizes and shapes. The software extracts a binary mask of the whole body of the arthropod from the image. From the mask, a number of traits describing phenotypic similarity were extracted. These were either related to body shape or size. The body shape was characterized by outline of the body segments (excluding appendages) and the circularity index, which describes body roundness (Herrera-Navarro et al., 2013). Outline was drawn following the straightening of the binary mask of each specimen in line with the body axis (Figure S5). Forty evenly distributed points were placed along the body length axis, and the distance from each of these points to the body edge was measured. As for size traits, the total body length and the thicknesses of each of four pairs of appendages (antennae and legs for insects, legs for spiders) were estimated. The thickness of each appendage was measured as the perpendicular distance of at the center of femur, tibia, and tarsus length.

#### Phylogeny

To study the co-evolution of traits, we used phylogenetic comparative methods. We constructed a truncated phylogeny including all mimetic species. The phylogenetic relationships among species at higher than species level were taken from existing phylogenies that were published most recently. Specifically, we used Misof et al. (2014) for the phylogeny of insect orders, McKenna et al. (2019) for the phylogeny of beetles, Johnson et al. (2018) and Jung and Lee (2012) for the phylogeny of Hemiptera, and Wheeler et al. (2017) for the phylogeny of spiders. The relationships at lower than genus level were reconstructed using new molecular data: For 36 species, sequences of the COI molecular marker were available from GenBank, and 34 species were bar-coded *de novo*.

All the material used for DNA analysis was preserved in pure ethanol. DNA was extracted using E.Z.N.A. Tissue DNA Kit (OMEGA BIO-TEK) following the manufacturer’s protocol. Individual specimens were rinsed in PBS buffer, placed in sterile tubes, and incubated overnight at 56°C with proteinase K. PCRs (total volume = 20 μL) were performed using barcoding primers for COI by Folmer et al. (1994). Amplified products were purified using the QIAquick PCR Purification Kit (QIAGEN). Sequencing was carried out with BigDye Terminator ver.3.1 (Applied Biosystems, Foster, CA) on an ABI 3100 genetic analysis sequencer (Perkin-Elmer Applied Biosystems, Norwalk, CT). All sequences were assembled and edited in SEQUENCHER 4.8 (Gene Codes Corporation, Ann Arbor, MI). GenBank accession numbers for the sequences are listed in Table S2. All sequences were aligned using MAFFT version 7 (Katoh and Standley, 2013) on the MAFFT server (http://mafft.cbrc.jp/alignment/server/). The method (L-INS-I) was automatically selected by the software according to the size of sequences being aligned. The resulting alignments were visually inspected and manually refined in MEGA 7 (Kumar et al., 2016), where necessary. The final molecular matrix containing 70 taxa was rooted using *Scorpio maurus*, with two *Euscorpio* species as outgroup*.*

To evaluate the best fit model for the model-based analyses, the third positions of COI were treated as separate partitions. Both partitions were evaluated in MrModeltest v.2.2 (Nylander, 2004) using both hierarchical likelihood ratio tests (hLRTs) and the Akaike Information Criterion (AIC). For both partitions we used GTR + Г + I as the best fitting evolutionary model (Rodriguez et al., 1990) for Bayesian inference. Bayesian inference was implemented in MrBayes version 3.2.6 (Huelsenbeck and Ronquist, 2001), and carried out on the CIPRES computer cluster (Cyber-Infrastructure for Phylogenetic Research; San Diego Supercomputer Center, Miller et al., 2010), with nucmodel = 4by4, ngen = 20mil, samplefreq = 1000, nruns = 2, and nchains = 4. Burn-in was set to 30%. All parameters were unlinked across partitions. The convergence of the runs was assessed by checking the potential scale reduction factor (PSRF) values of each parameter (in all cases, approaching 1.000) and the SD of split frequencies (<0.01) in MrBayes (Figure S9).

### Quantification and statistical analysis

Four movement traits were first scaled and transformed to be compared using MANOVA with a nested design. Velocity and mobility were transformed by square-root, whereas proportions were angularly transformed to approach normal distribution and homoscedasticity. Then we analyzed each trait separately using linear mixed models (LME) from the nlme package (Pinheiro et al., 2021) following appropriate transformation, to take into account the hierarchical structure of the data and to estimate the variance components of random effects (Pekár and Brabec, 2012). The fixed effect was the mimetic type (ant, control, and mimic), whereas the id of each mimetic triple (mimic-ant-control) and the taxonomic group (classified to order or family) were nested random effects. The taxonomic group contained eight arbitrary levels, so that each level was composed of a similar number of species to obtain balanced group design matrix: Corinnidae (Corinnidae and Phrurolithidae), Gnaphosidae, Salticidae (excluding *Myrmarachne* species), *Myrmarachne* species, Zodariidae, other spiders (Cyrtaucheniidae, Philodromidae, Theridiidae, Thomisidae, Titanoecidae), Hemiptera, and other insects (Mantodea, Coleoptera, Hymenoptera, Diptera) (see Table 1).

Twelve measures of appendage thickness (four appendages each having three segments) were combined and subjected to MANOVA with a nested design. Then the measurements were averaged and compared among the mimetic type using LME with similar settings as above. Both body size and appendage thickness were square-root transformed before analyses to homogenize their variance and approach normal distribution.

The circularity index, as a measure of shape, was analyzed using LME with similar settings as above.

To compare color among species in the triplet, we estimated the average vector of reflectance values for each species from the standardized scale. Then the Euclidean distance among vectors of mimics, ants, and control species was calculated. Similarly, to compare body shape, we first estimated the average outline vector per species standardized to the maximum, and the Euclidean distance among the vectors for mimics, ants and control species was calculated. The color and shape distances were then separately subjected to LME to study the difference/differences between ant-mimic and mimic-control pairs. The mimetic pair was the random effect.

#### Comparative analysis

We used the Euclidean distances (above) as estimates of the mimetic accuracy in each trait between each mimic species and its ant model. The movement category included four traits (velocity, movement, mobility, angular velocity), size category included two traits (total body and sum of leg thicknesses), and shape category include two traits (circularity and outline). The Euclidean distances within each category were summed and divided by the number of traits. As the distances were measured at different scales, we standardized them by scaling (divided by SD). Generalized least squares (GLS), with a covariance matrix based on the truncated phylogeny, was used to study the mutual relationships among the distances of four mimetic categories of traits and among all traits. As a covariance matrix, we used Pagel’s, Brownian motion, and Martin’s from the ape package (Paradis, 2006). Pagel’s covariance matrix usually had the smallest AIC, thus it was preferred. An ANCOVA model with two taxonomic groups, insects and spiders, as levels of a factor was fitted to each pair of mimetic trait categories. Pagel’s λ was used to measure phylogenetic signal (Münkemüller et al., 2012). All analyses were performed within R (R Core Team, 2021).

## Data Availability

• Data have been deposited at Mendeley Data and are publicly available as of the date of publication. DOIs are listed in the Key resources table. • All original code is available in this article’s supplemental information. • Any additional information required to reanalyze the data reported in this article is available from the lead contact on request.

## Supplemental information

Document S1. Figures S1–S9 and Tables S1 and S2

Data S1. Code used in the data analyses in R, related to Quantification and statistical analysis
