# Supplementary material for: Mimetic accuracy and co-evolution of mimetic traits in ant-mimicking species
Source: iScience. 2022 Sep 14;25(10):105126. doi: 10.1016/j.isci.2022.105126 (PMC9515603; doi:10.1016/j.isci.2022.105126)
Supplement: Document S1. Figures S1–S9 and Tables S1 and S2 [file mmc1.pdf]

iScience, Volume *25*

Supplemental information

Mimetic accuracy and co-evolution of mimetic traits in ant-mimicking species

Stano Pekár, Martina Martišová, Andrea Špalek Tóthová, and Charles R. Haddad

Table S1. List of sites (continent, locality) and habitat types where mimics, ants, and control species were collected. The numbers correspond to those in Table 1. Related to Table 1.

No.	Continent	Locality	Habitat
1.	Africa	South Africa, Ndumo Game Reserve	Bush foliage
2.	America	Central America, Costa Rica, La Selva Biological Station	Litter
3.	Africa	South Africa, Ndumo Game Reserve	Forest litter
4.	Africa	South Africa, Blooemfontein	Grassland
5.	Europe	Czechia, Hodonín, Pánov	Grassland
6.	Europe	Czechia, Senorady	Forest floor
7.	Europe	Czechia, Brno	Tree trunks
8.	Europe	Czechia, Lednice	Tree trunks
9.	Europe	Czechia, Pohořelice	Tree trunks
10.	Europe	Portugal, Mertola	Ground
11.	Europe	Portugal, Castro Marim	Ground
12.	Europe	Portugal, Mértola	Ground
13.	Europe	Czechia, Senorady	Leaf litter
14.	America	Central America, Costa Rica, La Selva Biological Station	Tree foliage
15.	Europe	Czechia, Brno	Grass
16.	Europe	Czechia, Pohansko	Tree trunks
17.	Africa	South Africa, Ndumo Game Reserve	Tree trunks
18.	Australia	NSW, North Ryde	Tree trunks
19.	Europe	Czechia, Steblová	Leaf litter
20.	Australia	QLD, Cairns, Botanical garden	Tree trunks
21.	Australia	QLD, Cairns	Bush foliage
22.	Europe	Czechia, Nosislav	Vine foliage
23.	Asia	Thailand, Sakaerat Silvicultural Research Station	Tree foliage
24.	Europe	Portugal, Serpa	Grassland
25.	Asia	Israel, Sede Boker	Stony desert
26.	Europe	Portugal, Moura	Leaf litter
27.	Asia	Israel, Mashabbim	Sands
28.	Europe	Czechia, Brno	Leaf litter
29.	Asia	Israel, Arad	Stony desert
30.	Africa	Breeding culture	
31.	Europe	Czechia, Dolní Kounice	Grass
32.	Europe	Czechia, Dolní Kounice	Leaf litter
32.	Europe	Czechia, Dolní Kounice	Tree foliage
33.	Australia	NSW, Blue Mountains	Tree trunks
34.	Europe	Czechia, Neslovice	Grass

Table S2. List of 70 taxa and their DNA accession (GenBank) numbers which were used in the phylogenetic analysis. Numbers in italics were species sequenced for this study. Species are arranged alphabetically within the class. Related to Figure 8.

Species	Accession number
Arachnida	
Amyciaea sp.	*OM321380*
Apochinomma formicaeforme	*OM321390*
Corcovetella galianoae	*OM321372*
Corinnomma semiglabrum	*OM321388*
Castianeira rica	*OM321383*
Castianeira sp.	MK154201
Euryopis episinoides	MH673872
Euscorpius alpha	KC496023
Euscorpius sicanus	HM418289
Heliophanus flavipes	MZ628546
Kima variabilis	*OM321389*
Leptorchestes berolinensis	KX039227
Liophrurillus flavitarsis	*OM321392*
Mazax pax	*OM321400*
Merenius alberti	*OM321381*
Mexcala elegans	MH673867
Micaria beaufortia	*OM321382*
M. formicaria	*OM321387*
M. fulgens	MH630633
M. micans	KX537201
M. sociabilis	*OM321374*
M. subopaca	KY269129
Myrmapana costaricaensis	*OM321384*
Myrmarachne erythrocephala	KX980382
M. formicaria	KP648564
M. helensmithae	KT364794
M. ichneumon	*OM321391*
M. kitale	*OM321401*
M. laurentina	*OM321402*
M. luctuosa	KX980381
M. lulengana	*OM321379*
M. macleayana	*OM321377*
M. marshalli	*OM321393*
M. russellsmithi	*OM321403*
M. smaragdina	KT364797
M. uvira	*OM321386*
Phrurolithus festivus	MT607868
Pulchellodromus bistigma	*OM321404*
Scorpio maurus	KF997875
Sylligma ndumi	*OM321373*
Synageles venator	KX536853
Synemosyna sp.	*OM321385*

Titanoeca spominima	*OM321399*
Trygetus sexoculatus	*OM321376*
Zodarion alacre	MT607949
Z. cyrenaicum	MH673876
Z. germanicum	MF919378
Z. luctuosum	*OM321375*
Z. nitidum	MF919379
Z. rubidum	MF919377
Insecta	
Alydus calcaratus	*OM321398*
Anthelephila pedestris	*OM321394*
Daerlac nigricans	KX980385
Eurymela sp.	MK055894
Gelis sp.	MN675021
Globiceps flavomaculatus	KM022304
Himacerus mirmicoides	*OM321397*
Lachnus roboris	LT600394
Micrelytra fossularum	*OM321396*
Myrmecoris gracilis	MZ631885
Myrmoplasta mira	*OM321405*
Palaeostigus palpalis	*OM321407*
Pilophorus perplexus	MF939009
Pithanus maerkelii	MZ629748
Raglius alboacuminatus	KM023067
Rhyparochromus vulgaris	KM022966
Sepsis thoracica	*OM321395*
Sphodromantis lineola	EF383804
Systellonotus triguttatus	MZ630165
Tentyrina orbiculata	*OM321406*

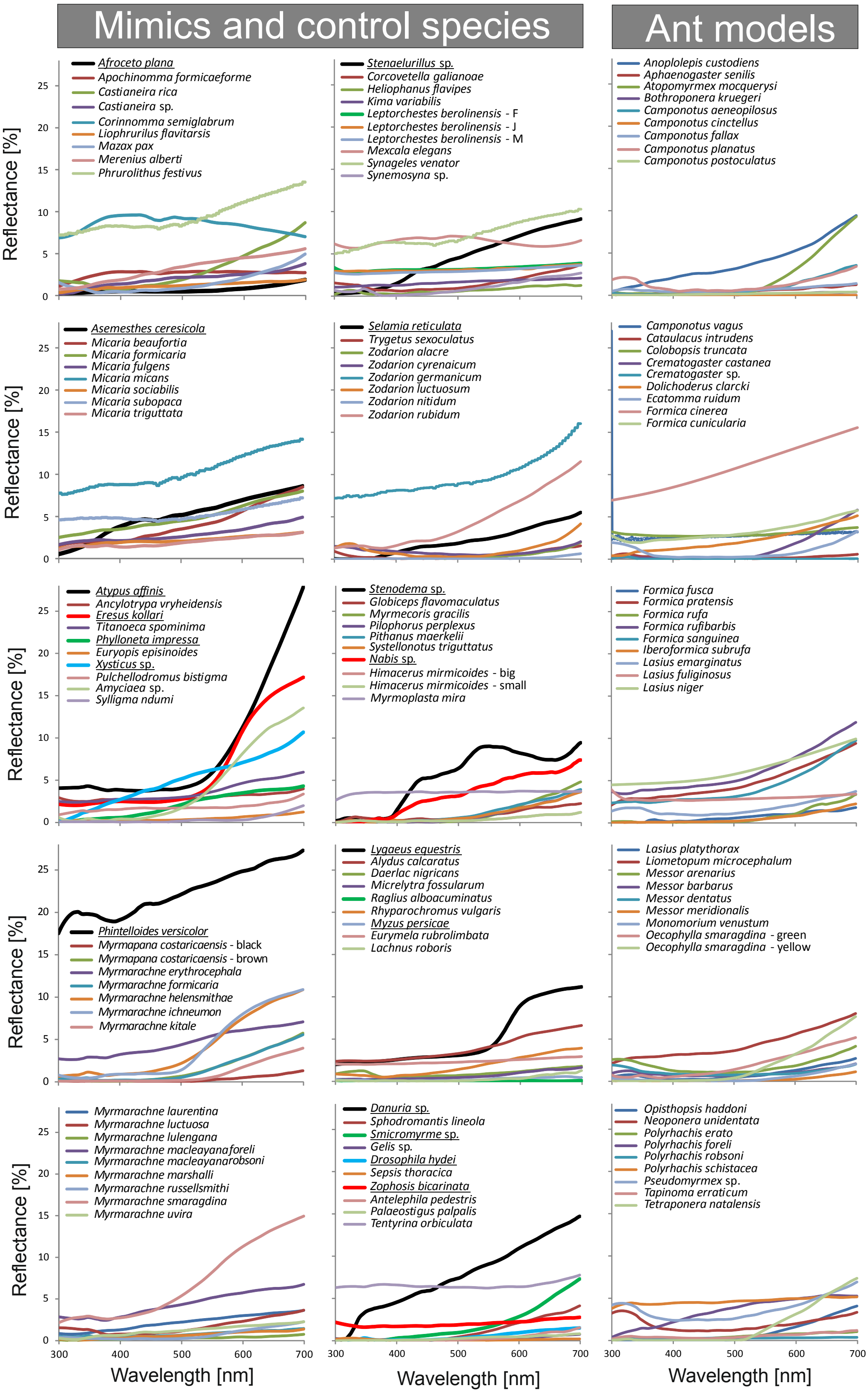

Figure S1. Mean reflectances of anterior body part (head+thorax or prosoma) of mimetic species, control species (underlined), and ant models. Related to Figure 7.

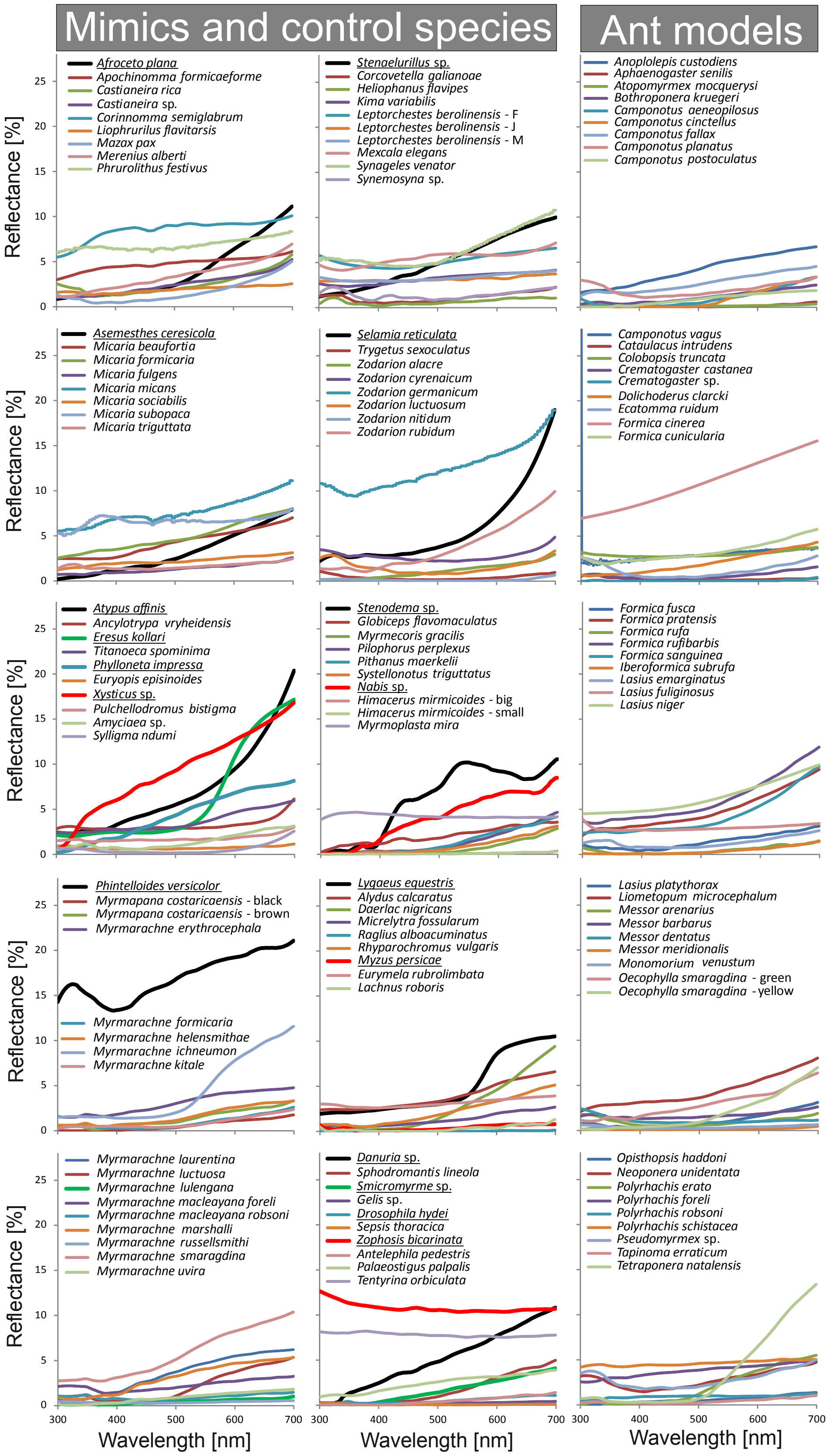

Figure S2. Mean reflectances of posterior body part (opisthosoma or gaster) of mimetic species, control species (underlined), and ant models. Related to Figure 7.

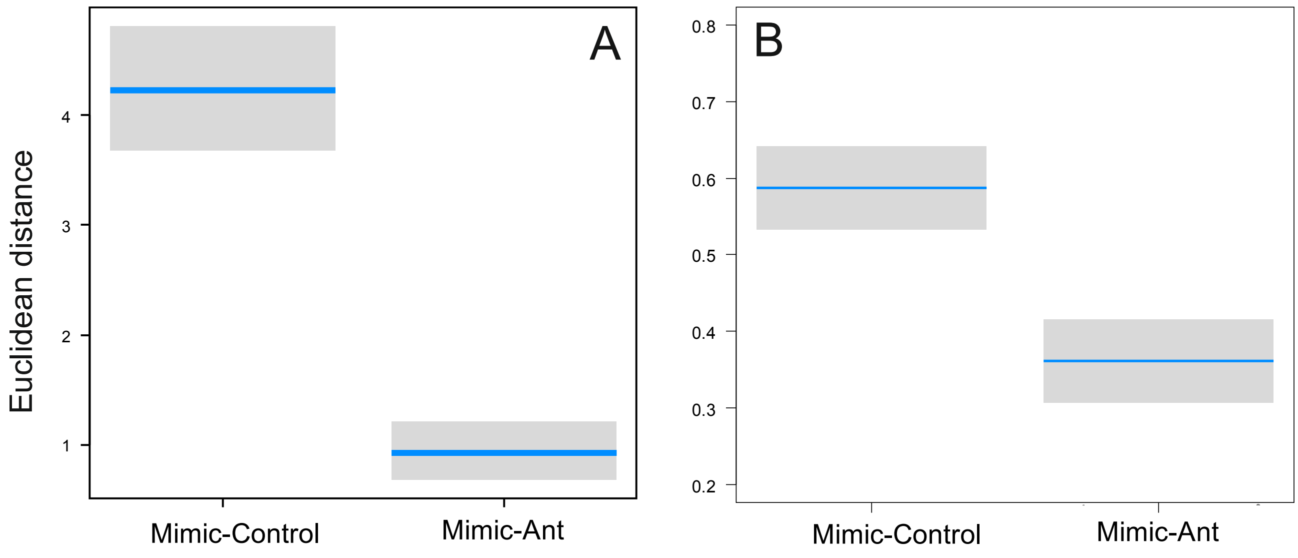

Figure S3. Comparison of the Euclidean distance in reflectance of the dorsal body (anterior and posterior parts combined) (A) and in body outline (B) between mimic-control and mimic-ant pairs. Blue lines are estimated means, grey bars are 95% confidence intervals of the mean. Related to Figure 7.

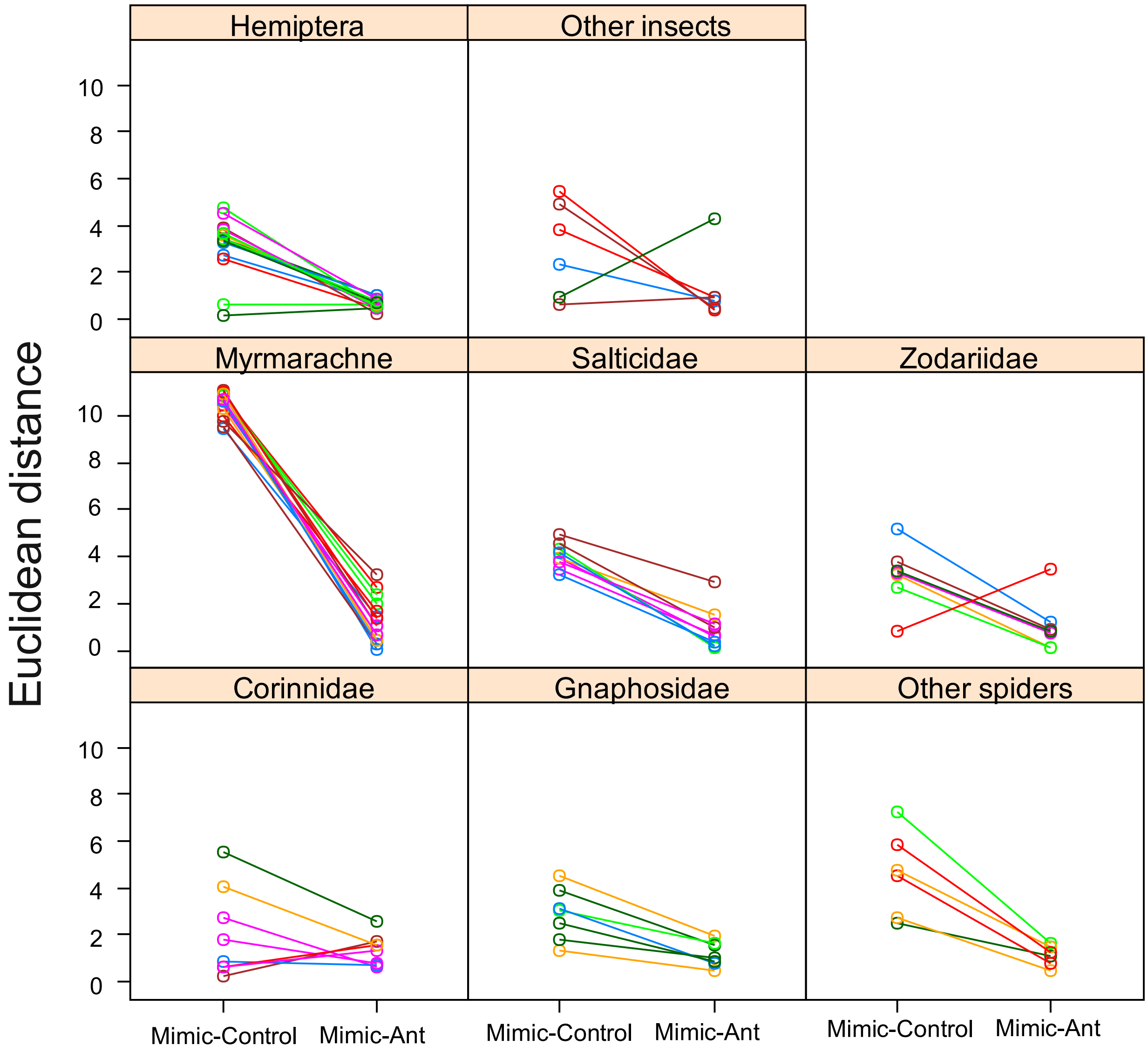

Figure S4. Comparison of mean Euclidean distances in reflectances between mimic-control and mimic-ant pairs. Every pair is represented by a different colour within a taxonomic group. For the definition of taxonomic groups, see Methods. Related to Figure 7.

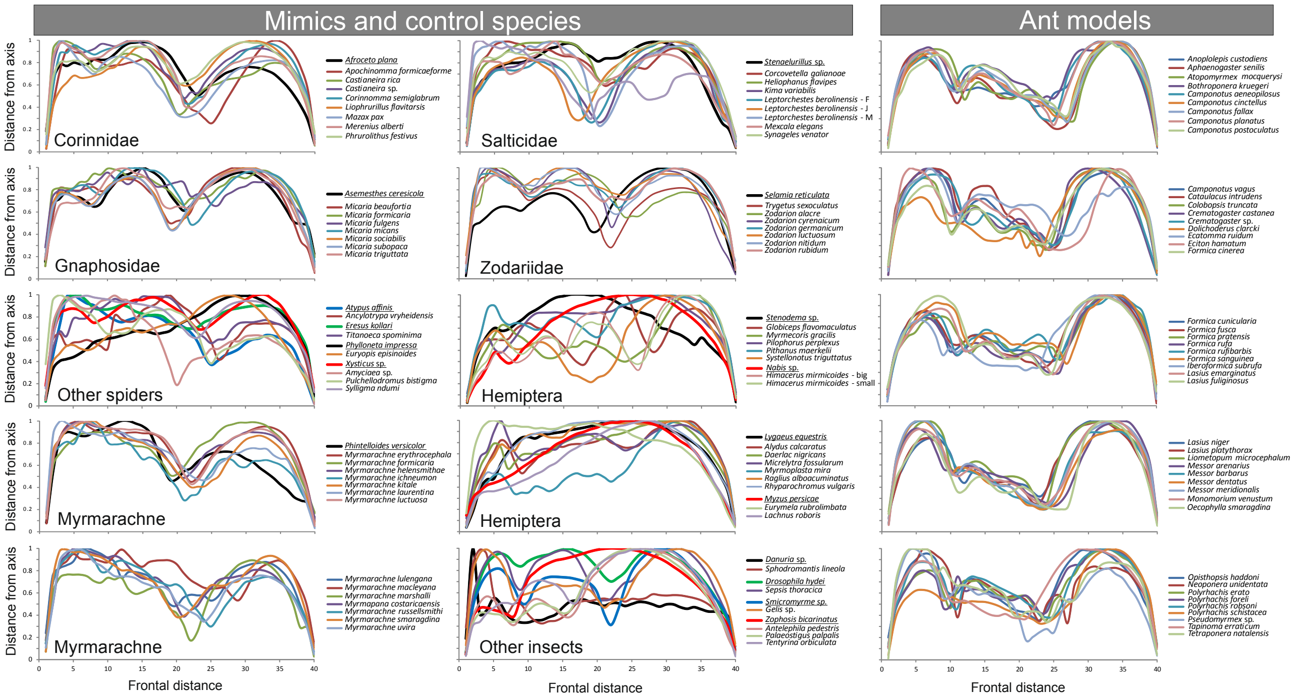

Figure S5. The outlines represent the right half of the body along the longitudinal body axis. Outlines are scaled along both axes. Related to Figure 7.

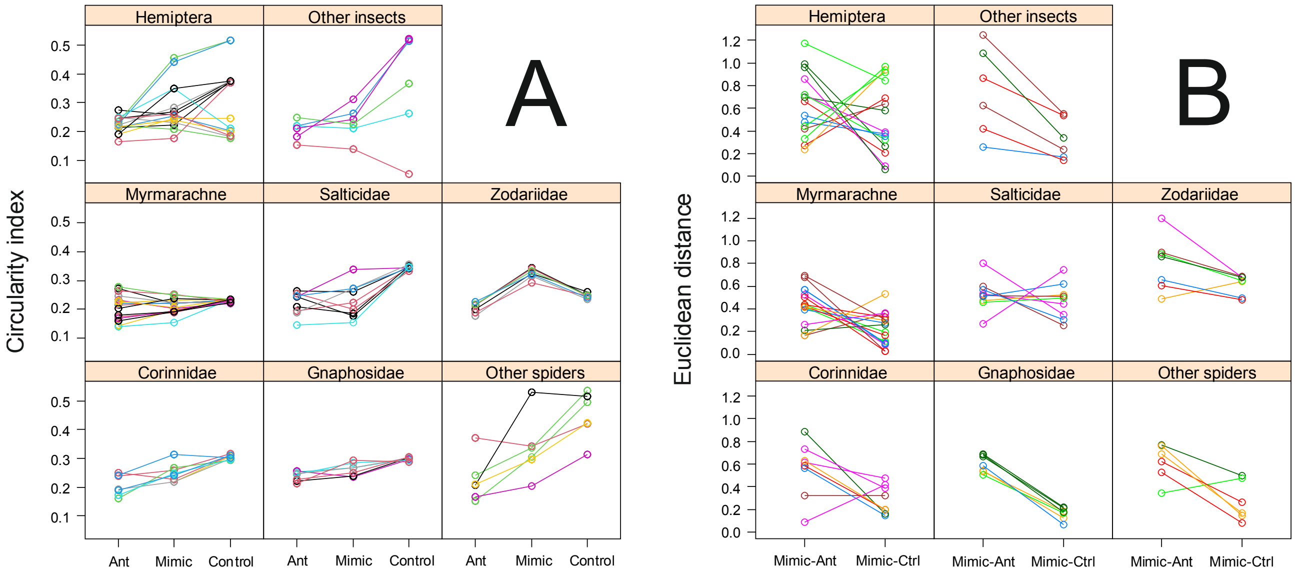

Figure S6. Comparison of the mean of two shape parameters, circularity index (A) and distance in body outline (B) between mimic-ant and mimic-control pairs. Every triplet (A) or pair (B) is represented by a different colour within a taxonomic group. For the definition of taxonomic groups, see Methods. Related to Figure 7.

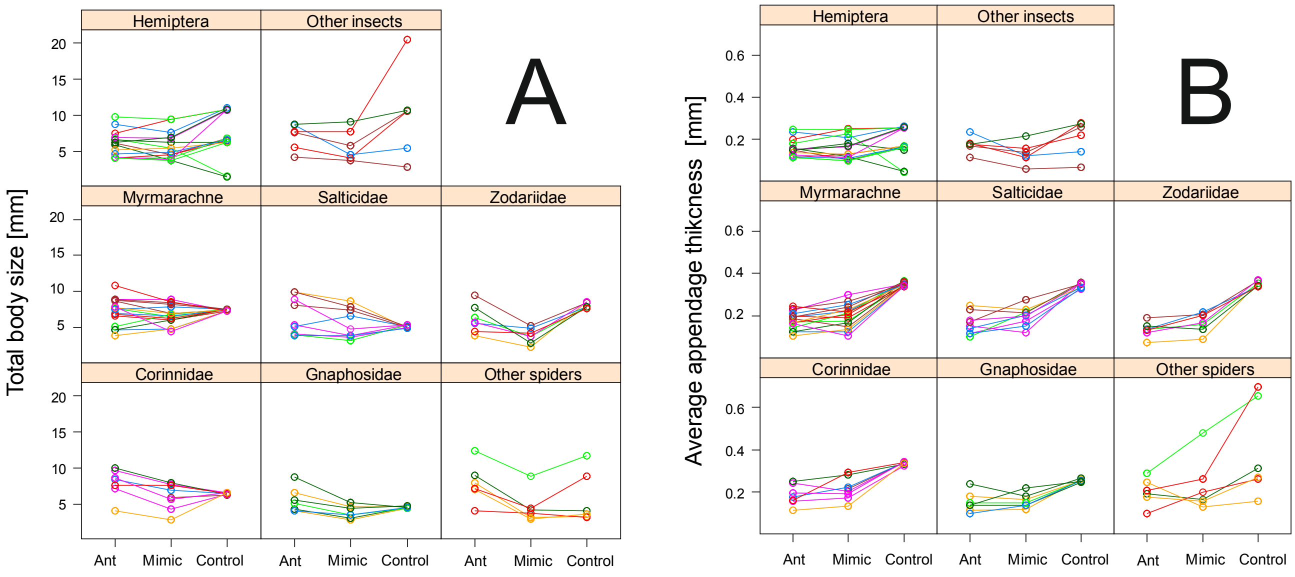

Figure S7. Comparison of two size parameters, mean total body length (A) and mean leg thickness (B), among mimics, ants, and control species. Every triplet is represented by a different colour within a taxonomic group. For the definition of taxonomic groups, see Methods. Related to Figure 5.

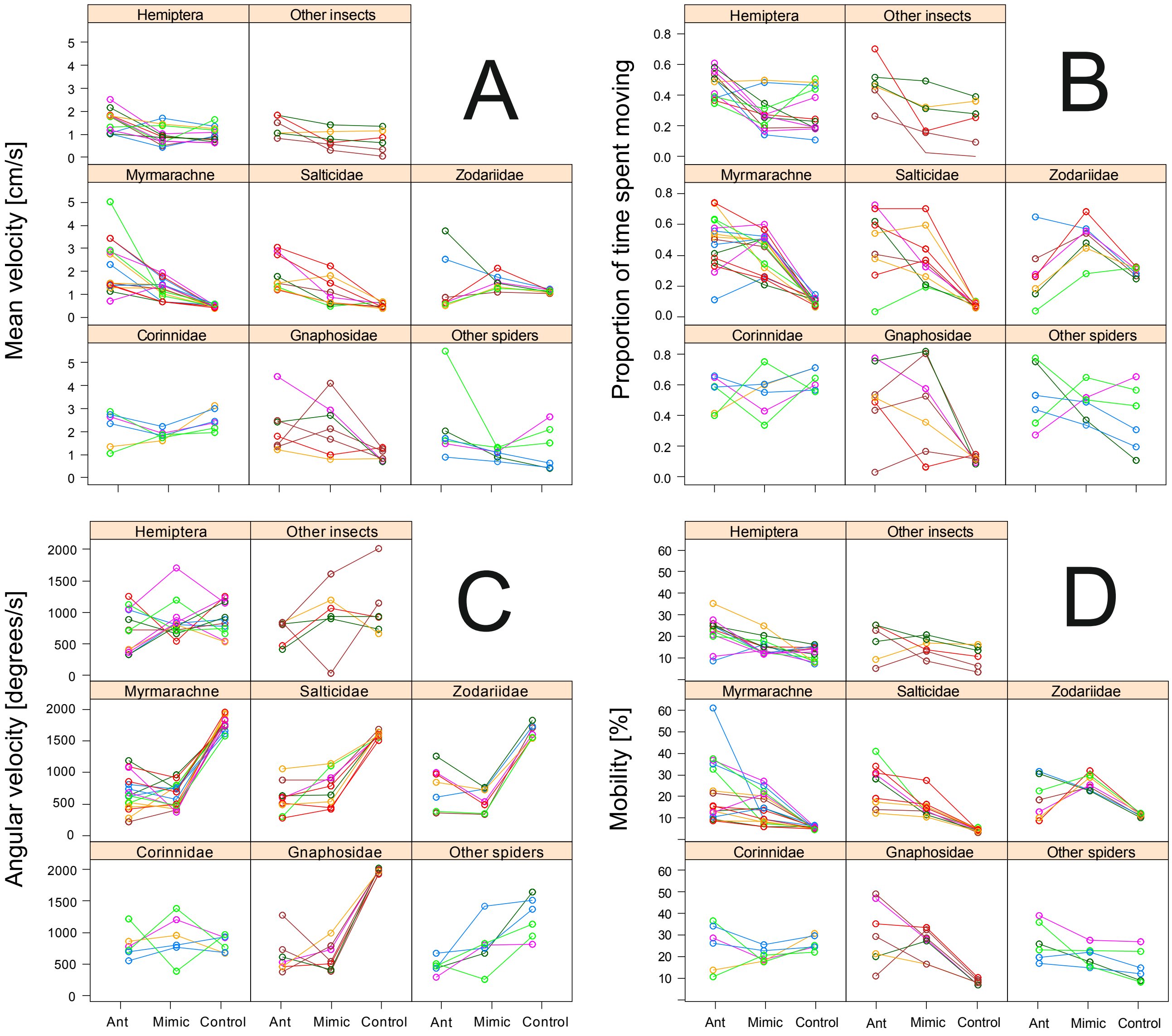

Figure S8. Comparison of four movement parameters, mean velocity (A), mean proportion of movement (B), mean angular velocity (C), and mean percentage of mobility (D), among mimics, ant, and -mimetic control species. Every triplet is represented by a different colour within a taxonomic group. For the definition of taxonomic groups, see Methods. Related to Figure 6.

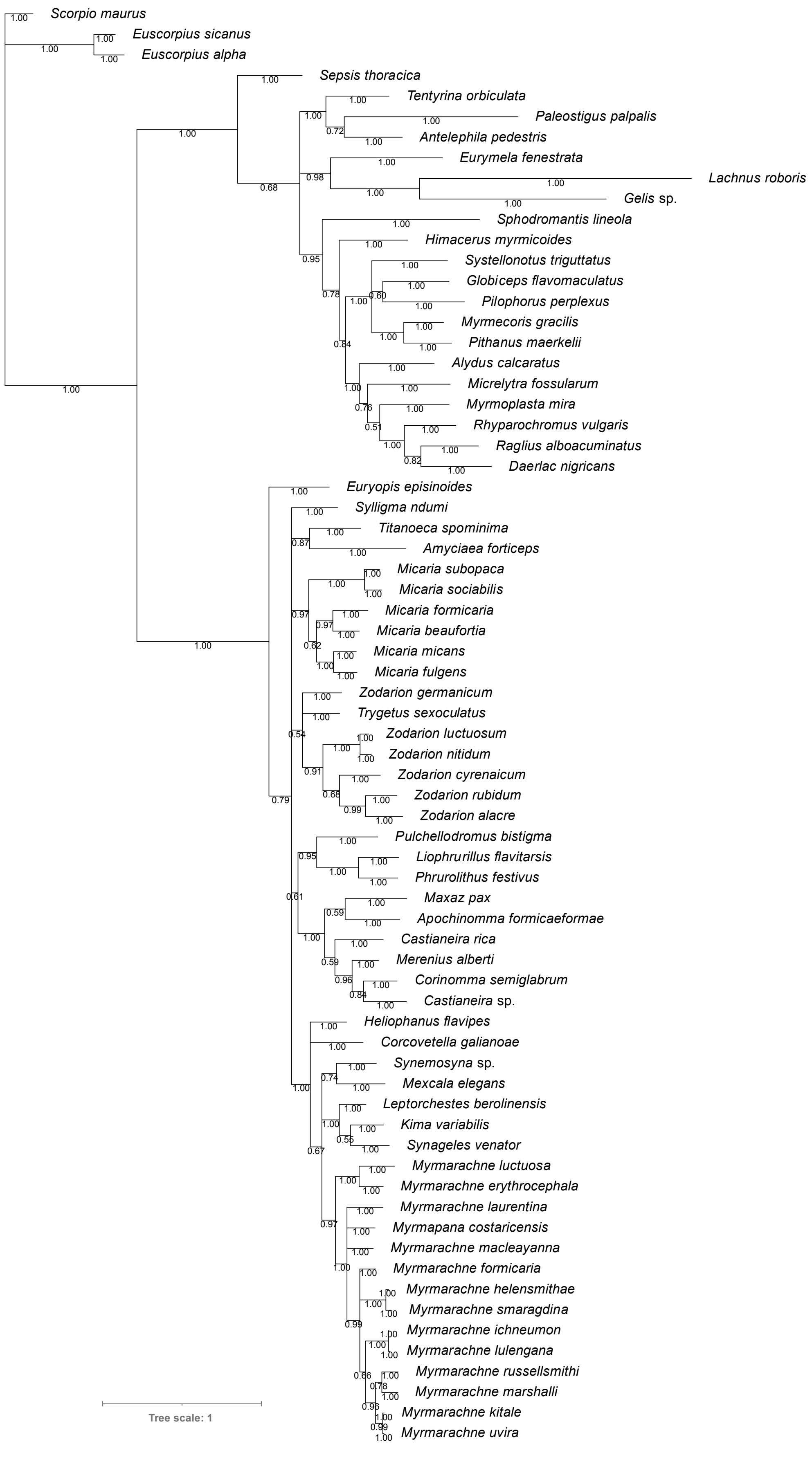

Figure S9. Phylogenetic tree including all mimetic species studied. Values below branches are the posterior probabilities of the Bayesian inference. Related to Figure 8.
