# Supplementary material for: Mimetic accuracy and co-evolution of mimetic traits in ant-mimicking species
Source: iScience. 2022 Sep 14;25(10):105126. doi: 10.1016/j.isci.2022.105126 (PMC9515603; doi:10.1016/j.isci.2022.105126)
Supplement: Data S1. Code used in the data analyses in R, related to Quantification and statistical analysis [file mmc2.docx]

Code used in the data analyses in R, related to Quantification and statistical analysis.

**Analysis of colour**

library(nlme)

*dcolo = Euclidean distance of reflectance measurements (a300-b699) between pairs (mimic vs model and mimic vs control)

m1a<-lme(dcolo~factor(type),random=~1|order/pair)

anova(m1a)

summary(m1a)

**Analysis of shape**

library(nlme)

m1a<-lme(circularity~factor(type),random==~1|order/pair)

anova(m1a)

summary(m1a)

*dshape = Euclidean distance of body outline measurements (a1-a40) between pairs (mimic vs model and mimic vs control)

m1b<-lme(dshape~factor(type),random=~1|order/pair)

anova(m1b)

summary(m1b)

**Analysis of sizes**

all<-cbind(total.body,app.1.proximal,app.1.medium,app.1.distal,app.2.proximal,app.2.medium,app.2.distal,app.3.proximal,app.3.medium,app.3.distal,app.4.proximal,app.4.medium,app.4.distal)

m1<-lm(all~factor(type)/factor(pair))

anova(m1)

library(nlme)

m1a<-lme(sqrt(total.body)~factor(type),random==~1|order/pair)

anova(m1a)

summary(m1a)

legs<-( app.1.proximal+app.1.medium+app.1.distal+app.2.proximal+app.2.medium+app.2.distal+app.3.proximal+app.3.medium+app.3.distal+app.4.proximal+app.4.medium+app.4.distal)/12

m1b<-lme(sqrt(legs)~factor(type),random=~1|order/pair)

anova(m1b)

summary(m1b)

**Analysis of movement**

vel<-scale(sqrt(velocity))

mov<-scale(asin(sqrt(time.spent.moving/100)))

ang<-scale(angular.velocity)

mob<-scale(sqrt(mobility))

anova(lm(cbind(ang,mob,vel,mov)~factor(type)/factor(pair)))

library(nlme)

m1a<-lme(vel~type,random=~1|order/pair)

anova(m1a)

summary(m1a)

m1b<-lme(mov~type,random=~1|order/pair)

anova(m1b)

summary(m1b)

m1c<-lme(ang~type,random=~1|order/pair)

anova(m1c)

summary(m1c)

m1d<-lme(mob~type,random=~1|order/pair)

anova(m1d)

summary(m1d)

**Trait coevolution**

*dvel = Euclidean distance between mean values of velocity measurements of mimics and models

*dmov = Euclidean distance between mean values of time spent moving of mimics and models

*dang = Euclidean distance between mean values of angular velocity between of mimics and models

*dmob = Euclidean distance between mean values of mobility of mimics and models

move<-(scale(dvel)+scale(dmov)+scale(dang)+scale(dmob))/4

*colo = Euclidean distance between mean values of reflectances of mimic and model

*dleg = Euclidean distance between mean values of leg thickness of mimics and models

*dtot = Euclidean distance between mean values of total body of mimics and models

size<-(scale(dleg)+scale(dtot))/2

*dcir = Euclidean distance between mean values of circularity measurements of mimics and models

*dprof = Euclidean distance between mean values of body outline of mimics and models

shape<-(scale(dcir)+scale(dprof))/2

distance<-rbind(colo,size,shape,move)

m1<-lme(distance~factor(trait),random=~1|order/pair)

anova(m1)

library(ape)

tree<-read.tree(text="((Ancylotrypa.vryheidensis,(Euryopis.episinoides,(Titanoeca.spominima,((Trygetus.sexoculatus,(Zodarion.germanicum,((Zodarion.luctuosum,Zodarion.nitidum),(Zodarion.cyrenaicum,(Zodarion.alacre,Zodarion.rubidum))))),((Amyciaea.sp,Sylligma.ndumi),(((Liophrurillus.flavitarsis,Phrurolithus.festivus),((Micaria.subopaca,Micaria.sociabilis),((Micaria.formicaria,Micaria.beaufortia),(Micaria.triguttata,(Micaria.micans,Micaria.fulgens))))),((Apochinomma.formicaeforme,(Mazax.pax,(Castianeira.rica,(Merenius.alberti,(Corinnomma.semiglabrum,Castianeira.sp))))),(Pulchellodromus.bistigma,((Heliophanus.flavipes,Corcovetella.galianoae),((((Leptorchestes.berolinensis.F,Leptorchestes.berolinensis.J),Leptorchestes.berolinensis.M),(Kima.variabilis,Synageles.venator)),((Synemosyna.sp,Mexcala.elegans),((Myrmarachne.erythrocephala,Myrmarachne.luctuosa),((Myrmapana.costaricaensis.br,Myrmapana.costaricaensis.bl),((Myrmarachne.macleyana.foreli,Myrmarachne.macleyana.robsoni),(Myrmarachne.laurentina,((Myrmarachne.helensmithae,Myrmarachne.smaragdina),(Myrmarachne.formicaria,((Myrmarachne.ichneumon,Myrmarachne.lulengana),((Myrmarachne.kitale,Myrmarachne.uvira),(Myrmarachne.marshalli,Myrmarachne.russellsmithi)))))))))))))))))))),(Sphodromantis.lineola,(((Lachnus.roboris,Eurymela.rubrolimbata),((((Systellonotus.triguttata,Pilophorus.perplexus),(Globiceps.flavomaculatus,(Myrmecoris.gracilis,Pithanus.maerckeli))),(Himacerus.mirmicoides.big,Himacerus.mirmicoides.small)),(((Alydus.calcaratus,Micrelytra.fossularum),Myrmoplasta.mira),(Daerlac.nigricans,(Raglius.alboacuminatus,Rhyparochromus.vulgaris))))),(Gelis.sp,((Palaeostigus.palpalis,(Tentyrina.orbiculata,Antelephila.pedestris)),Sepsis.thoracica)))));")

tree1<-compute.brlen(tree,1)

rownames(dat)<-tree1$tip.label

dat1<-dat[tree1$tip.label,]

cp<-corPagel(0.5,phy=tree1,form=~spe)

library(nlme)

m1a<-gls(colo~1,cor=cp,data=dat1,na.action=na.omit)

m1a

m1b<-gls(move~1,cor=cp,data=dat1,na.action=na.omit)

m1b

m1c<-gls(shape~1,cor=cp,data=dat1,na.action=na.omit)

m1c

m1d<-gls(size~1,cor=cp,data=dat1,na.action=na.omit)

m1d

bm<-corBrownian(phy=tree1,form=~spe)

ma<-corMartins(phy=tree1,form=~spe)

m2a<-gls(colo~move*order,cor=cp,data=dat1,na.action=na.omit)

anova(m2a)

m3a<-gls(colo~move*order,cor=bm,data=dat1,na.action=na.omit)

anova(m3a)

m4a<-gls(colo~move*order,cor=ma,data=dat1,na.action=na.omit)

anova(m4a)

m2b<-gls(colo~shape*order,cor=cp,data=dat1,na.action=na.omit)

anova(m2b)

m2c<-gls(colo~size*order,cor=cp,data=dat1,na.action=na.omit)

anova(m2c)

m2d<-gls(shape~move*order,cor=cp,data=dat1,na.action=na.omit)

anova(m2d)

m2e<-gls(size~move*order,cor=cp,data=dat1,na.action=na.omit)

anova(m2e)

m2f<-gls(shape~size*order,cor=cp,data=dat1,na.action=na.omit)

anova(m2f)
